# A trait‐based integration of competition and abiotic tolerance underlies plant success

**DOI:** 10.1111/nph.71465

**Published:** 2026-07-23

**Authors:** Jan Douda, Jana Doudová, Přemysl Král, Jan Häusler, Anežka Holeštová, Leandro Eusebio, Jiří Květoň, Alena Havrdová

**Affiliations:** ^1^ Faculty of Environmental Sciences Czech University of Life Sciences Prague Kamýcká 129 Prague 165 00 Czech Republic; ^2^ Faculty of Science University of South Bohemia Branišovská 1760 České Budějovice 370 05 Czech Republic

**Keywords:** competition–abiotic tolerance trade‐off, competitive effect, competitive response, functional traits, local dominance, niche breadth, phylogenetic conservatism, relative growth rate

## Abstract

- Understanding what determines species' ecological success is a central challenge in ecology. Competitive ability and abiotic tolerance are expected to be strong predictors of species success, yet theory predicts a trade‐off between them. Since some traits are phylogenetically conserved whereas others are evolutionarily labile, species may have the potential to combine competitive ability with abiotic tolerance.
- We conducted a garden experiment involving 23 wetland species under two contrasting hydrological regimes and quantified performance measures to assess species responses to competition and abiotic conditions. We also measured structural, physiological, and morphological traits, and related both to species success, quantified as realized niche breadth and local dominance across 19 000 vegetation plots.
- Niche breadth and local dominance were most strongly associated with competitive effect, and to a lesser extent with abiotic tolerance. The most successful species expressed traits associated with a phylogenetically conservative persistence–stature functional dimension (high LDMC, tall stature, and deep roots) together with traits of evolutionarily labile acquisitive physiology (low δ^13^C, high stomatal conductance, and elevated leaf nitrogen), indicating high gas exchange and sustained carbon acquisition while trading off rapid growth.
- Our findings show that competitive strength and abiotic tolerance can co‐occur, helping explain why certain evolutionary lineages dominate wetlands.

## Graphical abstract

Competitive effect and abiotic tolerance show positive associations with species' niche breadth and local dominance.

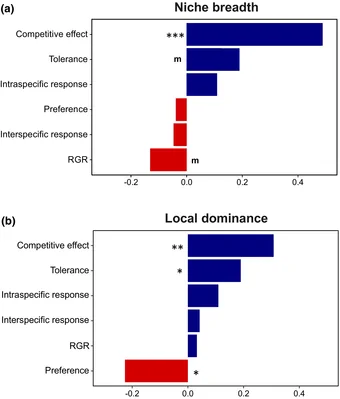

## Shrnutí

- Pochopení toho, proč jsou některé druhy úspěšnější než jiné, patří mezi základní otázky ekologie. Za hlavní předpoklady ekologického úspěchu jsou považovány schopnost obstát v konkurenci a odolnost vůči nepříznivým podmínkám prostředí. Mezi těmito vlastnostmi se však předpokládá kompromis (trade‐off). Skutečnost, že jednotlivé funkční znaky se liší mírou své evoluční konzervovanosti, však vytváří potenciál pro současný vývoj obou strategií.
- V zahradním experimentu jsme pěstovali 23 druhů mokřadních rostlin ve dvou kontrastních hydrologických režimech a hodnotili jejich schopnost obstát v konkurenci i tolerovat rozdílné podmínky prostředí. Současně jsme změřili řadu strukturálních, morfologických a fyziologických znaků a zkoumali jejich vztah k ekologickému úspěchu jednotlivých druhů, vyjádřenému šířkou realizované ekologické niky a lokální dominancí ve více než 19 000 vegetačních snímcích.
- Šířka ekologické niky i lokální dominance byly nejsilněji korelovány s kompetičním efektem, zatímco tolerance k abiotickým podmínkám s nimi souvisela rovněž pozitivně, avšak slaběji. Nejúspěšnější druhy kombinovaly fylogeneticky konzervované znaky spojené s investicí do struktur (vysoké LDMC, vysoký vzrůst a hluboký kořenový systém) se znaky evolučně labilními, odrážejícími akvizitivní fyziologii (nízké hodnoty δ^13^C, vysokou průduchovou vodivost a vysoký obsah dusíku v listech). Tato kombinace svědčí o intenzivní výměně plynů a dlouhodobě vysoké schopnosti získávat uhlík, avšak za cenu nižší rychlosti růstu.
- Naše výsledky ukazují, že vysoká kompetiční schopnost a tolerance k nepříznivým podmínkám prostředí se nemusí vzájemně vylučovat. Jejich kombinace může vysvětlovat, proč některé evoluční linie rostlin dominují mokřadním ekosystémům.

## Introduction

A central question in ecology, with broad implications for invasion and evolutionary biology, is what determines species success in natural communities (Rejmanek & Richardson, 1996). Trait‐based approaches have long sought to explain species success, but their predictive power remains limited (Pyšek *et al*., 2009; Doudová *et al*., 2017; Funk *et al*., 2017; van der Plas *et al*., 2020). These limitations point to the need to link functional traits to direct measures of ecological performance, particularly competitive ability and abiotic tolerance (Traveset & Richardson, 2020; Gioria *et al*., 2023). Because these measures are more closely linked to the realized niche, they may provide more informative proximate indicators of species success (Slatyer *et al*., 2013; Carscadden *et al*., 2020). Several studies have used the maintenance of plant performance as a proxy for abiotic tolerance across broad sets of species under different experimental conditions (Keddy *et al*., 1994; Wang *et al*., 2010), yet whether such performance‐based measures predict species success in natural communities remains unclear.

The ‘competition–abiotic tolerance trade‐off’ is a longstanding assumption in ecological theory (Grime, 1977; Tilman & Pacala, 1993; Martin & Ghalambor, 2023), but the mechanisms underlying it remain poorly understood (Liancourt *et al*., 2005). In the classical view, competitive ability is closely linked to rapid growth under favourable conditions, implying an inevitable trade‐off with abiotic tolerance (Grime & Hunt, 1975). By contrast, Tilman (1988) argued that tolerant species may also be strong competitors because the same traits that enhance persistence under resource scarcity can confer competitive advantages. This alternative perspective is consistent with recent field evidence showing a positive relationship between drought tolerance and resistance to competition (Mount *et al*., 2024).

A more complete understanding of how growth, competition, and abiotic tolerance interact may arise from examining how these traits align with fundamental axes of plant function. The acquisitive–conservative resource‐use spectrum (Wright *et al*., 2004) and the persistence–stature axis (Westoby *et al*., 2002; Díaz *et al*., 2016) together capture how plants balance growth, robustness, and resource allocation. Integrating these functional axes with performance measures provides a mechanistic framework for explaining why some species combine strong competitiveness with high abiotic tolerance, whereas others rely on rapid growth (Liu *et al*., 2025). Traits underlying persistence and stature are typically phylogenetically conserved, whereas acquisitive physiological traits tend to be evolutionarily labile (Westoby *et al*., 2002; Ackerly, 2009; Cavender‐Bares *et al*., 2009). Recognizing these phylogenetic differences in trait conservatism is critical for mechanistic insight into whether high competitive ability and abiotic‐tolerance trade‐off or can be combined.

Variation in local dominance and species' realized niche reflects how environmental stress and competition jointly shape plant strategies across heterogeneous landscapes (Bell *et al*., 2000; Schellenberger Costa *et al*., 2018). In wetland habitats, strong hydrological fluctuations create steep contrasts in water availability – the major axis of abiotic stress – while maintaining high resource supply and dense neighbourhood interactions (Douda *et al*., 2018; Doudová & Douda, 2020). Consequently, plants experience contrasting stressors, from drought and desiccation in seasonally dry patches to oxygen deprivation during prolonged saturation. These contrasting conditions select for divergent adaptive strategies even among closely related species (Moor *et al*., 2017). Such hydrological extremes promote local dominance of species with contrasting functional traits and abiotic‐tolerance mechanisms, structured by trade‐offs between drought resistance, anaerobic metabolism, and resource acquisition (Silvertown *et al*., 1999; Voesenek & Bailey‐Serres, 2015; Pan *et al*., 2019). At the same time, species' physiological tolerance to drought and flooding largely determines their distributions across wetland habitats (Bell *et al*., 2000; Comita & Engelbrecht, 2014), yet how these stress axes interact with competition remains poorly understood. To address this, we experimentally quantified competitive ability as two complementary components: the competitive effect, describing a species' capacity to suppress the growth of neighbours, and the competitive response, its ability to tolerate competition from them. We also accounted for intraspecific competition, which reflects density‐dependent self‐limitation within populations (Hart *et al*., 2018). From an evolutionary perspective, species experiencing strong self‐limitation may compensate by occupying a broader range of habitats and thus maintaining a wider realized niche (Giller, 1984).

By linking experimentally measured traits of 23 common Central European wetland plant species with their distribution patterns across more than 19 000 stratified vegetation plots, we quantify species success at two distinct ecological scales: the breadth of realized niche, inferred from species' occurrence across the full range of wetland habitats; and local dominance, reflecting species performance within sites. Here, species success is used in a strictly operational sense to refer to these two dimensions, rather than to general evolutionary or ecological fitness. To relate these field‐based metrics to experimentally derived traits, we further assess two axes of abiotic adaptation: abiotic tolerance, expressed as the difference in species' performance between dry and waterlogged conditions, and abiotic preference, reflecting relative performance under these regimes and indicating specialization to contrasting hydrological extremes. This integrative framework provides a means to test whether key measures of ecological performance and their trade‐offs – particularly those involving growth, abiotic tolerance, and competitive ability – are associated with species success and whether these patterns are phylogenetically conserved or have evolved independently across lineages. Specifically, because competition was quantified only under productive, high‐water conditions, our design tests whether competitive ability covaries with abiotic tolerance across contrasting hydrological regimes, but does not test whether competitive hierarchies shift between dry and waterlogged conditions.

## Materials and Methods

### Selected species and experimental setup

We selected 23 vascular plant species based on their phytosociological affinity to wetland communities (Supporting Information Methods S1, Chytrý, 2011). Wetland vegetation is typically dominated by graminoids, particularly the diverse and functionally important genus *Carex*, but also includes forbs that play key roles; therefore, species selection was stratified to reflect this structural composition. We included 15 graminoids (5 grasses and 10 sedge‐like species) and eight forbs (see Table S1 for species list and Fig. S1 for phylogeny). All species are perennials.

Between May and September 2023, we conducted a mesocosm experiment in the experimental garden of the Faculty of Life Sciences, Czech University of Life Sciences Prague (50°07′51.5″N, 14°22′16.0″E). The site lies in the European temperate zone with a subcontinental climate of warm, dry summers and cold winters. From May to September 2023, the mean daily temperature was 18.1°C, *c*. 1.3°C above the 1961–2023 average. Total rainfall was 225 mm, *c*. 51 mm below the long‐term mean (Fig. S2).

A total of 1221 individuals were cultivated in 765 PVC tubes (7.4 l; 370 mm height; 160 mm diameter; Martoni S.p.A., San Vito al Tagliamento, Italy) placed in 60 tanks of two sizes (600 × 600 × 350 mm and 1300 × 600 × 350 mm). Tubes were open at the bottom to allow water saturation but restricted root growth outside the tube. Tubes were filled with a 1 : 1 mixture of commercial soil and sand, while surrounding areas in tanks were kept soil‐free and filled with open water. The design included three growth scenarios: (1) waterlogged conditions without interspecific competition (high‐water treatment; *n* = 178 tubes), (2) desiccation simulating wetland drying without interspecific competition (drought treatment; *n* = 131 tubes), and (3) waterlogged conditions with interspecific competition in all pairwise species combinations (competition treatment; *n* = 456 tubes). In the noncompetitive treatment, plants were grown in the centre of each tube, whereas in the competitive treatment, individuals were planted 5 cm apart around the centre. Detailed parameters of water‐level manipulation are provided in Methods S2. Experimental treatments were randomly assigned to tanks, and the positions of individual species within each treatment were also randomized to avoid spatial bias (see Fig. S3 for details of the experimental setup). To ensure robust replication in the competition experiment, which involved *c*. three quarters of all cultivated individuals, we restricted competition to a single water regime. We chose the high‐water treatment, as it best represents typical wetland conditions and a more productive environment, where competitive effects are expected to be more pronounced (Douda *et al*., 2021). To balance the feasibility of this large‐scale experiment with the need for replication, we limited each species to six replicates under high‐water and drought treatments. In the competition treatment, each species pair was planted in two replicates. Due to variable germination success and mortality during establishment, the final number of plants per species differed (Table S1).

### Seed collection and plant establishment

For each species, fully mature seeds were collected from three natural wetland populations during the 2022 growing season. Seeds were pooled across populations before stratification to maintain genetic diversity. Germination was ensured in two ways: under controlled conditions in a climate chamber and under more natural temperature conditions outdoors, to maximize germination success across species (see Methods S1 for details on germination and seedling cultivation). In May 2023, similar‐sized seedlings were transplanted into experimental tubes placed in tanks. To ensure balanced representation across treatments and competition pairs, dead plants were replaced during the establishment phase lasting 4 wk (Table S1).

### Aboveground biomass, growth rate, and functional traits

To quantify changes in relative growth rates (RGRs) during the growing season, aboveground parts of each plant were measured seven times: at seedling transplantation, before the onset of experimental treatments (20 June), and subsequently at 2‐wk intervals until 7 September. We recorded a set of morphological traits known to correlate with biomass, including the number of ramets, plant height, leaf length, number of branches, and number of leaves, to estimate species‐specific aboveground biomass, as plant biomass could not be measured nondestructively (Table S2). Aboveground biomass (harvested at the end of the growing season and dried to constant weight) was used as the response variable, with trait sets serving as predictors in species‐specific biomass models. The resulting model equations (with trait coefficients, see Table S2) were used to predict aboveground biomass at all seven time points for each individual. These biomass predictions were used to model daily changes in species biomass. From the biomass trajectories, we calculated daily absolute growth rate (AGR) as the first derivative (i.e. daily change in biomass), and relative growth rate (RGR) as the AGR divided by the biomass at that time point (see Methods S3 for details). As an alternative proxy of RGR, we also repeated the analysis using plant height, which was measured at seven time points throughout the growing season in all species. However, height‐based RGR may be less suitable as a general proxy of growth in perennial, and especially clonal, species, where growth is to a large extent expressed laterally.

To characterize functional strategies of the studied species, we measured a suite of morphological, physiological, and chemical traits following standardized protocols (Table S3; Methods S4). Traits included leaf dry matter content (LDMC), specific leaf area (SLA), leaf element concentrations (C, N, P), carbon isotope composition (δ^13^C), stomatal conductance, stomatal fraction, plant height, maximum root length (MRL), root specific volume (RSV), and root mass fraction (RMF). Traits were measured for all individuals except analytically demanding traits (LDMC, SLA, δ^13^C, and leaf C, N, and P concentrations), which were determined for all drought and waterlogged replicates and for a random subset of six individuals per species in the competition treatment.

### Abiotic response and competitive ability

Three types of Bayesian models were fitted to quantify different components of species performance based on total plant biomass (sum of above‐ and belowground parts): (1) abiotic response (tolerance and preference), (2) competitive (inter‐ and intraspecific) response, and (3) competitive effect. Abiotic response models tested for the effect of water availability by comparing total plant biomass between drought and high‐water treatments. In these models, total plant biomass was the response variable and water treatment was the predictor. It was expressed in two ways: as preference, with positive values indicating higher biomass in dry conditions; and tolerance, calculated as the absolute value of treatment contrast. We note that these two metrics are not fully independent, as both are derived from the same biomass contrast between hydrological treatments.

Competitive response models assessed the impact of competition under high‐water conditions by comparing biomass between plants grown with competitors and those grown alone. Here, total plant biomass was the response variable and competition treatment (with vs without competitor) was the predictor. To quantify intraspecific response we used a parallel mesocosm experiment conducted in the same area in 2024 under permanently waterlogged conditions (see Methods S5 for details on the experiment and the estimation of intraspecific response). Competitive effect models quantified how each competitor species influenced the biomass of focal species under high‐water conditions by comparing the biomass of focal species grown with and without a given competitor. In these models, focal‐species biomass was the response variable and competitor identity/presence was the predictor. In the latter case, the identity of the focal species was included as a random effect to account for interspecific variation in baseline biomass; all other models contained fixed effects only. For each species, we also tested for tank effects using Bayesian mixed models. As these effects were negligible (individuals were randomly distributed across tanks), we proceeded with species‐specific Bayesian models that did not include tank as a random factor. All models were implemented in R using the rstanarm package with a Gaussian error distribution. Biomass data were log‐transformed when necessary to improve normality of residuals. We used weakly informative priors, four Markov chains, and 4000 iterations per chain, discarding the first 1000 iterations as warm‐up. Convergence was confirmed by Gelman–Rubin statistics (all *R̂* < 1.01), effective sample sizes (ESS > 2000), and visual inspection of trace plots. Model fit was evaluated using posterior predictive checks, and model quality was assessed via approximate leave‐one‐out cross‐validation with Pareto *k* diagnostics (flagging *k* > 0.7 as potentially influential observations). From the posterior distributions, we extracted treatment contrasts: the difference between drought and high‐water conditions for the abiotic response, between the biomass of plants grown in competition and those grown alone for the competitive response, and between the biomass of neighbouring species grown with a focal species and their biomass when grown alone for the competitive effect. To enable comparison among species differing markedly in biomass and biomass variance, posterior contrasts were standardized by dividing each contrast by the species‐specific SD of biomass across treatments, resulting in standardized posterior distributions. These standardized metrics should therefore be interpreted as relative species‐specific effect sizes, indicating how pronounced biomass shifts across treatments or competition contexts are relative to the typical variability of each species. Thus, a small absolute shift may still represent a strong response in a less variable species, whereas a larger shift may be less meaningful in a species with high intrinsic biomass variability. Species‐level estimates were represented by posterior medians, chosen for their robustness to skewed posterior distributions and extreme values.

Although each species pair was replicated only twice, the species‐level competition metrics used in the comparative analyses were based on all pairwise interactions of each focal species with the remaining species. Depending on establishment success, the number of observations contributing to species‐level estimates ranged from 15 to 58 per species (Table S1).

### Trade‐offs between competitive ability, abiotic response and growth rate

To explore potential trade‐offs among competitive ability, abiotic response, and growth rate, we performed phylogenetically informed regression analyses using phylogenetic generalized least squares (PGLS). Specifically, we tested whether species' competitive effect or response was related to their abiotic preference and tolerance, with alternative models also compared by corrected Akaike information criterion (AICc) and collinearity among predictors assessed using variance inflation factors (VIF). We also tested whether competitive and abiotic response metrics were associated with species' RGR. All models were calculated using caper R package, with Pagel's λ estimated by maximum likelihood. The phylogenetic tree was based on the complete Phylogeny of Central European flora (Durka & Michalski, 2012) and was pruned to include the full set of species used in our experiment. Depending on model fit, response variables were log‐transformed where necessary. We then used a sensitivity analysis to assess how posterior uncertainty affected the analyses by repeatedly resampling species‐level metrics from posterior distributions (*n* = 2000), refitting the PGLS models, and summarizing the resulting distributions of regression slopes.

To analyse multivariate structure and associations among functional traits and performance measures, we combined species‐level median trait values across treatments. Independent functional axes were identified using hierarchical clustering based on absolute Pearson correlations with complete linkage, complemented by estimates of Pagel's λ to assess phylogenetic conservatism of traits (R package phytools). A phylogenetically corrected PCA was then used to visualize functional trade‐offs among traits, revealing opposing directions of trait variation along major functional gradients. Finally, both standard Pearson correlations and phylogenetically corrected correlations derived from PGLS models were used to relate functional traits to species' competitive ability, RGR, and abiotic tolerance and preference. To assess potential nonindependence among functional traits, we additionally calculated pairwise Pearson correlations among all measured traits.

### Species success in natural communities

We used vegetation‐plot data (records of all plant species occurring at a site with estimates of their cover) from the Czech National Phytosociological Database (Chytrý & Rafajová, 2003) to quantify the breadth of the realized niche and the local dominance of 23 wetland plant species. All plots containing at least one target species were extracted and spatially stratified to decrease spatial autocorrelation and sampling bias (see Methods S6 for details). The spatial stratification resulted in a final dataset of 19 420 relevés, which were used in all subsequent analyses. Species niche breadth was quantified using the θ index (Zelený & Chytrý, 2019), calculated as multiplicative beta diversity with the R package theta. Higher θ values indicate broader realized niches. Local dominance was defined as the mean of species' proportional cover, calculated as its cover in a plot divided by the sum of all species covers in that plot, averaged across all plots where the species occurred. We related niche breadth (θ) and local dominance (log‐transformed) to six performance predictors using PGLS models, with alternative predictor combinations compared also by AICc. We also tested species occurrence within the Czech Republic, expressed as the percentage of occupied cells in the 5 × 3 arc‐minute grid of the Central European Basic Area (CEBA) distribution (Schönfelder, 1999), as another potential measure of species success. However, because it showed no significant relationships with any of the six predictors, it was not included in the main text of the article.

## Results

**Fig. 1:**
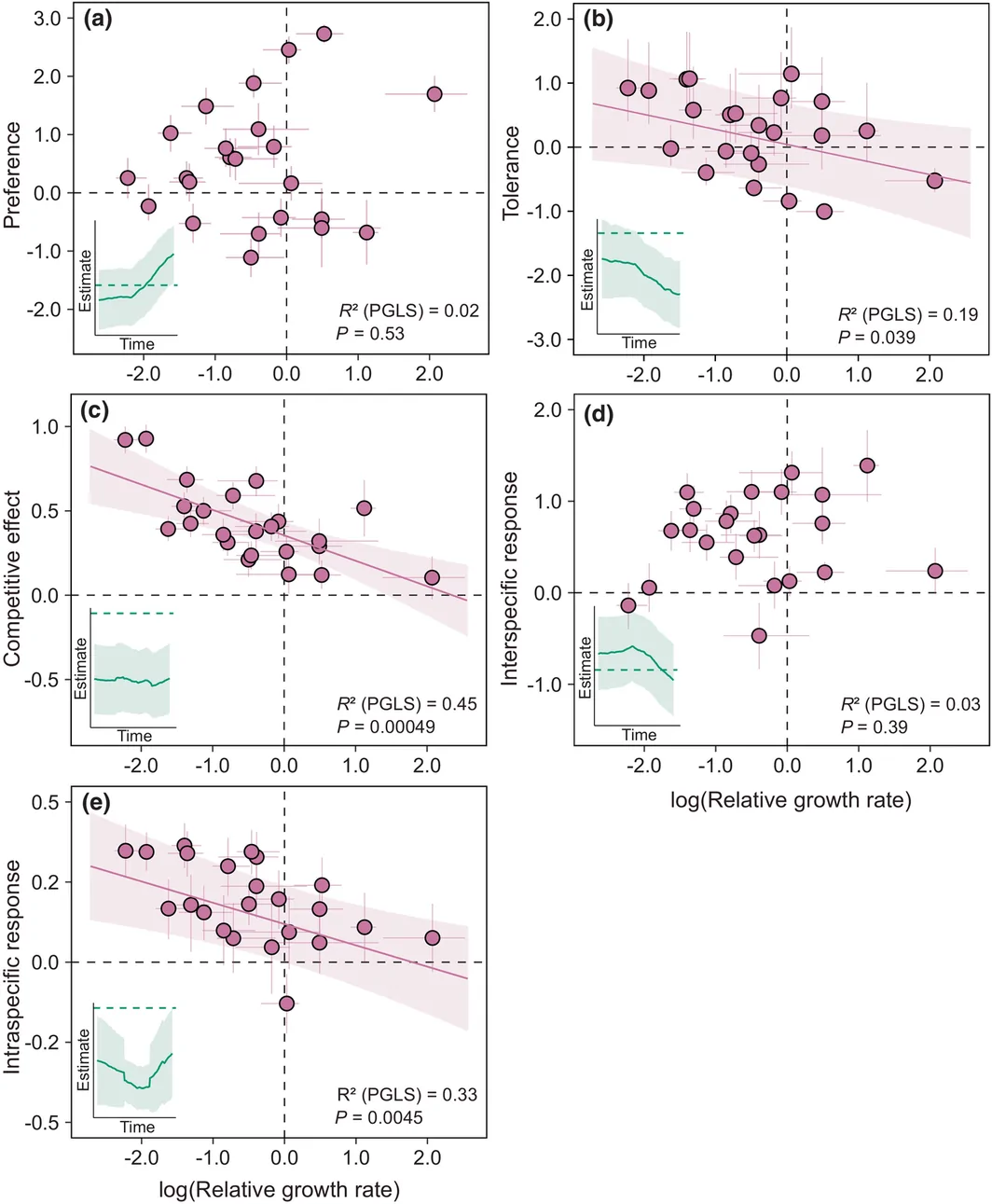
Relationships between relative growth rate (estimated based on plant biomass) and measures of abiotic preference (a), abiotic tolerance (b), competitive effect (c), interspecific response (d), and intraspecific response (e) in 23 wetland plant species. Points represent species‐level median estimates; error bars show 50% confidence intervals. Lines are phylogenetic generalized least squares (PGLS) fits with its 95% confidence band. The bottom‐left inset depicts the seasonal trajectory of the standardized PGLS slope (green line) with its 95% confidence band and the dashed green horizontal line indicates null slope.

We found a strong negative association between RGR (estimated based on plant biomass) and species' competitive ability. This relationship was observed for competitive effect (Fig. 1c; *R* ^2^ = 0.45, *P* < 0.001) and intraspecific competitive response (Fig. 1e; *R* ^2^ = 0.33, *P* < 0.01), but not for interspecific competitive response (Fig. 1d). A significant negative association was also found between RGR and abiotic tolerance (Fig. 1b; *R* ^2^ = 0.19, *P* = 0.039). All negative relationships were supported by posterior resampling, with 90%, 100%, and 96% of posterior refits retaining a negative slope for abiotic tolerance, competitive effect, and intraspecific response, respectively (Fig. S4B,C,E). The significance of these relationships remained unchanged when RGR was estimated from plant height (Fig. S5B,C,E). Competitive effect was positively correlated with intraspecific response (Fig. S6B; *R* ^2^ = 0.27, *P* = 0.012), with 89% of posterior refits retaining a positive slope (Fig. S7B). By contrast, interspecific response was not correlated with either competitive effect (Fig. S6A) or intraspecific response (Fig. S6C).

**Fig. 2:**
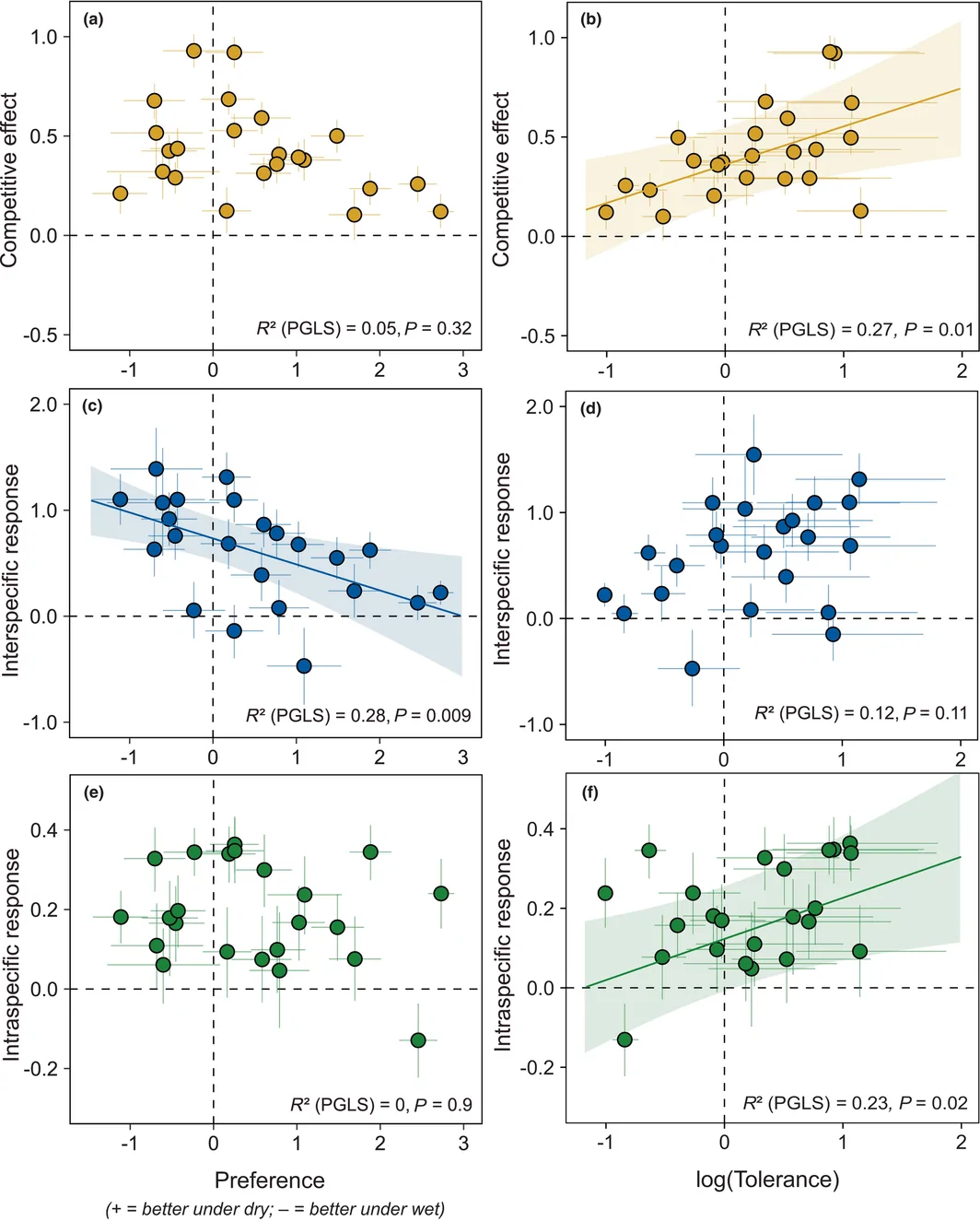
Relationships between measures of abiotic response (preference: (a, c, e); tolerance: (b, d, f)) and three aspects of competitive ability in 23 wetland plant species. Points represent species‐level median estimates; error bars show 50% confidence intervals. Lines are phylogenetic generalized least squares (PGLS) fits with its 95% confidence band.

Contrary to expectations of a trade‐off, more competitive species were also more abiotic tolerant, as indicated by positive relationships of abiotic tolerance with competitive effect (Fig. 2b; *R* ^2^ = 0.27, *P* = 0.01). No significant relationship was found between abiotic tolerance and interspecific response (Fig. 2d), whereas abiotic tolerance was positively related to intraspecific response (Fig. 2f; *R* ^2^ = 0.23, *P* = 0.02). We also found a significant negative association between species' preference for dry conditions and their interspecific response (Fig. 2c; *R* ^2^ = 0.28, *P* < 0.01), while other measures of competitive ability showed no relationship with environmental preferences (Fig. 2a,e). These patterns were further supported by PGLS model comparison, which identified tolerance‐only models as the best‐supported models for competitive effect and intraspecific response, and the preference‐only model as the best‐supported model for interspecific response (Table S4). Posterior refits retained the same slope direction for most significant relationships, although support was weaker for one case than for the others (62–99%; Fig. S8B,C,F).

**Fig. 3:**
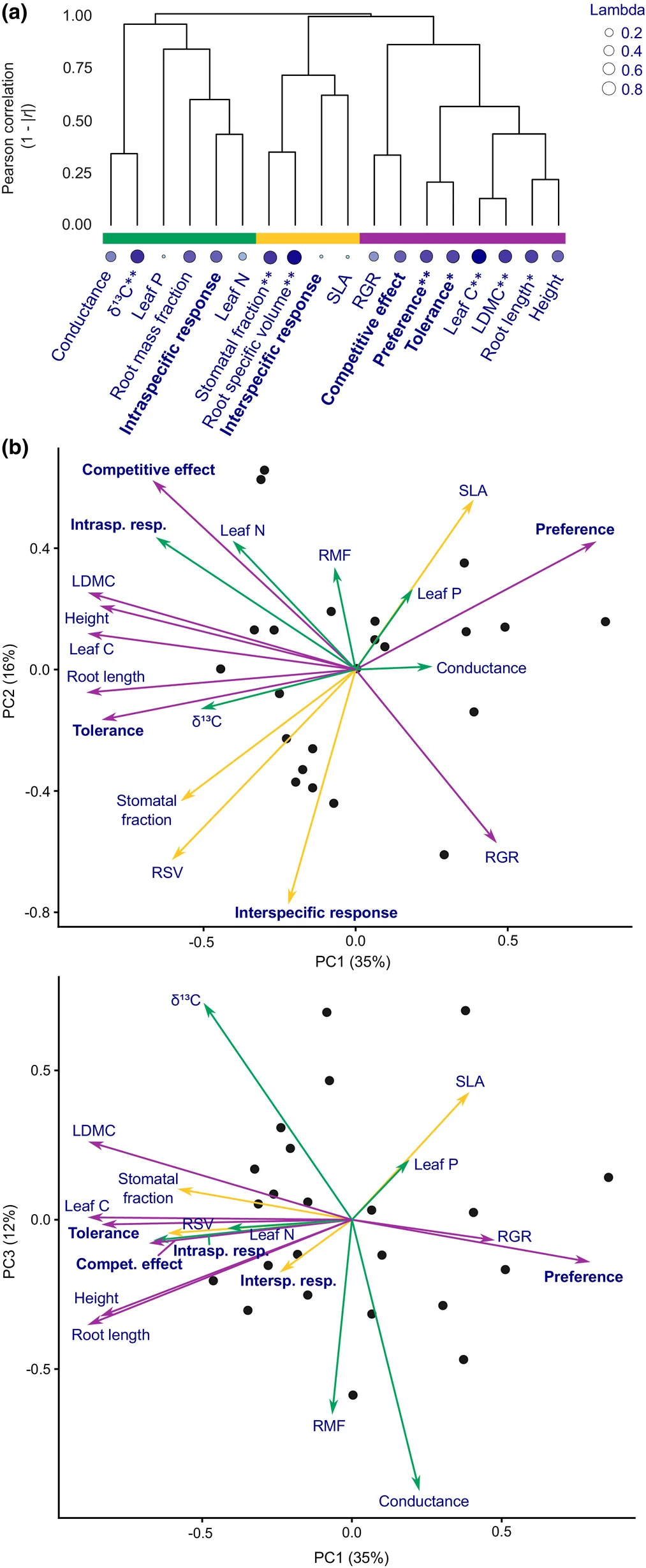
Relationships among functional traits, performance measures and their phylogenetic signal across 23 wetland plant species. (a) Distance‐based dendrogram of five performance measures (bold blue) and 13 functional traits (blue), constructed using absolute Pearson correlations and agglomerative complete linkage. The resulting three clusters are as follows: (1) physiological–acquisitive traits (green), including stomatal conductance (conductance), carbon isotope ratio (δ^13^C), leaf phosphorus content (leaf P), root mass fraction (RMF), intraspecific response and leaf nitrogen content (leaf N); (2) leaf–root acquisitive morphology (yellow) comprising stomatal fraction, specific leaf area (SLA), interspecific response and root specific volume (RSV); and (3) persistence–stature traits (violet) consisting of leaf carbon content (leaf C), leaf dry matter content (LDMC), abiotic preference (preference), abiotic tolerance (tolerance), maximum root length (MRL), plant height (height), relative growth rate (RGR), and competitive effect. Circle size and blue colour intensity indicate the strength of phylogenetic signal (Pagel's λ). Asterisks denote significance of the λ test: *, *P* < 0.05; **, *P* < 0.01. (b) The first three principal components (PCs) from the phylogenetically corrected principal component analysis (PCA) of 23 wetland plant species. Arrow tips indicate the loadings of the measured characteristics across species (Supporting Information Fig. S10).

The cluster analysis of trait–trait correlations identified three broad trait groupings among the 13 functional traits (Fig. 3a). For reference, these groupings are here labelled according to the predominant functional characteristics of the traits they include as physiological–acquisitive traits, leaf–root acquisitive morphological traits, and persistence–stature traits. The physiological–acquisitive group includes stomatal conductance, carbon isotope ratio (δ^13^C), leaf nitrogen (leaf N) and phosphorus (leaf P) content and RMF. The leaf–root acquisitive morphology group includes SLA, stomatal fraction and RSV. The persistence–stature group combines RGR, leaf carbon content (leaf C), LDMC, MRL, and plant height. PGLS analysis indicates significant conservatism for δ^13^C, stomatal fraction, RSV, leaf C, LDMC, and MRL (Fig. 3a). These broad trait groupings were reflected in the first three PCA axes, which together explained 62% of the variance (Fig. S9). PC1 was primarily associated with a persistence–stature spectrum, with species increasing in MRL, leaf C, LDMC and height along the axis (Figs 3b, S10A). PC2 was primarily associated with a leaf–root acquisitive morphology spectrum, contrasting species with high SLA against those with high RSV and stomatal fraction (Figs 3b, S10B). PC3 was primarily associated with a physiological–acquisitive spectrum, contrasting species with high δ^13^C against those with high stomatal conductance and RMF (Figs 3b, S10C).

**Fig. 4:**
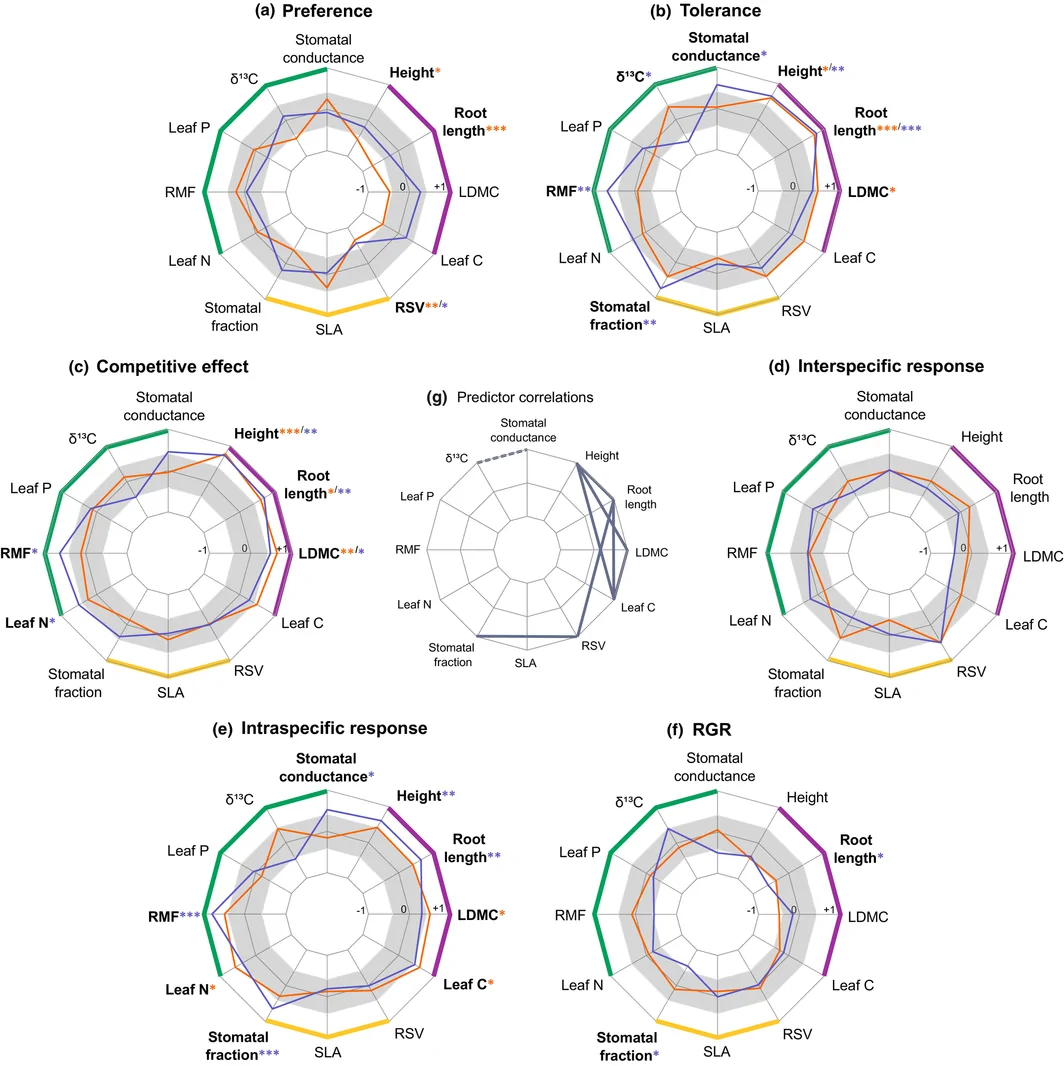
Functional trait correlates of abiotic response (a, b), competitive ability (c–e), and relative growth rate (f) among 23 wetland plant species. Orange lines show Pearson correlation coefficients ranging from −1 at the centre to 1 at the outer edge of each radar plot. Blue lines represent phylogenetically corrected correlations. The grey band indicates the 95% confidence interval under the null hypothesis of no correlation. Asterisks in the trait labels indicate statistically significant correlations after Benjamini–Hochberg correction for multiple testing, with orange stars denoting significance in Pearson correlation and blue stars denoting significance after the phylogenetic correction (*, *P* < 0.05; **, *P* < 0.01; ***, *P* < 0.001). Line colours correspond to ecological strategy clusters: green = physiological–acquisitive, yellow = leaf–root acquisitive morphology, violet = persistence–stature traits. Panel (g) summarizes pairwise Pearson correlations among functional traits. Lines connect trait pairs with strong correlations (|*r*| ≥ 0.6). Solid lines denote positive correlations, whereas dashed lines denote negative correlations. LDMC, leaf dry matter content; leaf C, leaf carbon content; RSV, root specific volume; SLA, specific leaf area; leaf N, leaf nitrogen content; RMF, root mass fraction; leaf P, leaf phosphorus content; δ^13^C, carbon isotope ratio.

In terms of performance measures, abiotic tolerance, abiotic preference, and intraspecific response were primarily associated with PCA1, whereas interspecific response was mainly associated with PCA2. Competitive effect was related to both PCA1 and PCA2, while no performance measure was clearly associated with PCA3 (Figs 3b, S10). Abiotic preference was negatively correlated with plant height, MRL, and RSV (Fig. 4a). Abiotic tolerance was positively correlated with stomatal conductance, stomatal fraction, plant height, MRL, LDMC, and the RMF, and negatively with δ^13^C (Fig. 4b). Competitive effect was positively related to most persistence–stature traits (plant height, MRL, and LDMC) as well as the RMF and leaf N (Fig. 4c). A similar pattern was observed for intraspecific response, which was positively correlated with stomatal conductance, plant height, MRL, LDMC, leaf C, leaf N, stomatal fraction, and the RMF (Fig. 4e). Interspecific response and RGR showed only weak correlations with functional traits (Fig. 4d,f). However, several traits were strongly intercorrelated (Fig. 4g; Tables S5, S6), especially within the persistence–stature group.

**Fig. 5:**
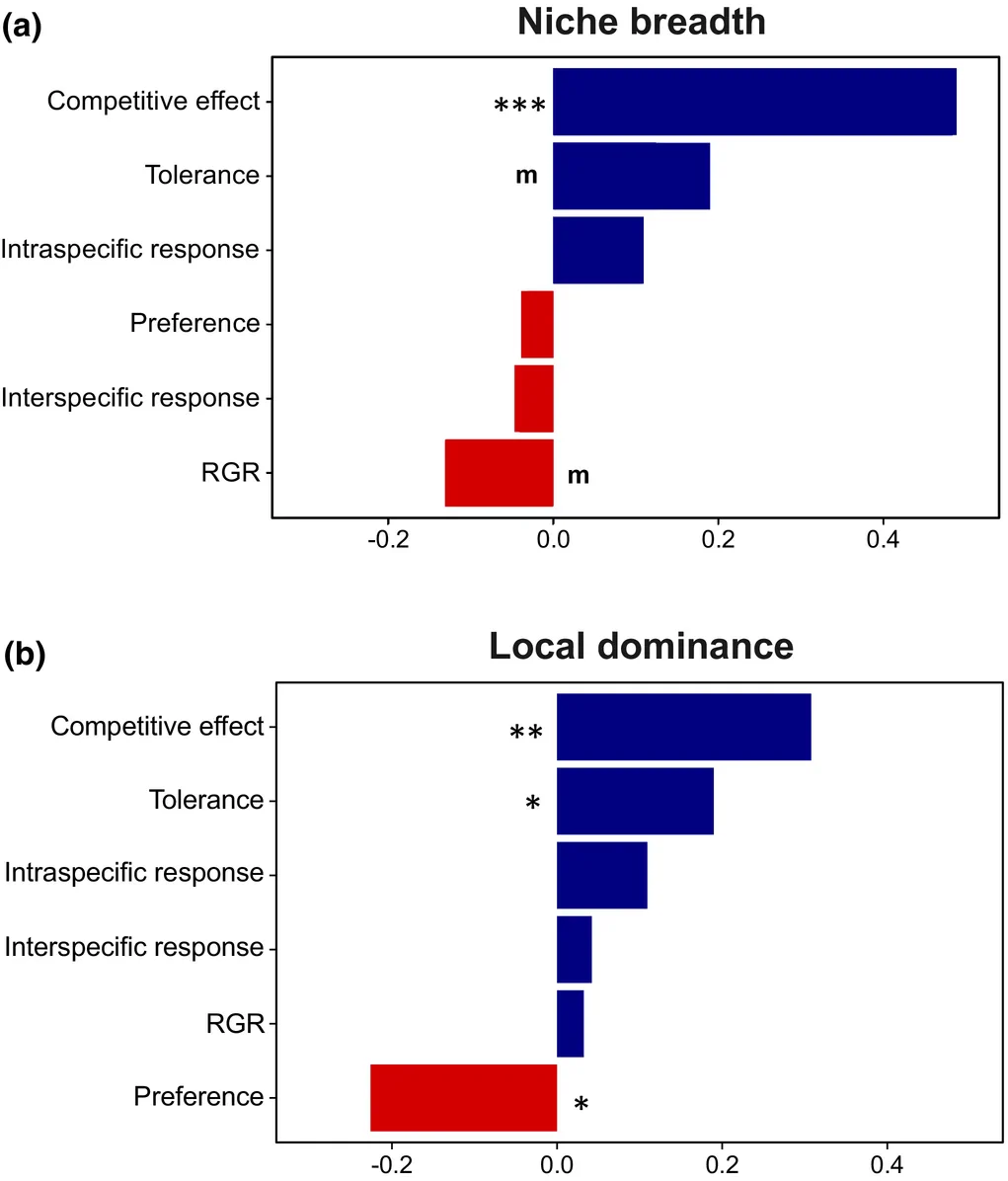
Niche breadth (θ) and local dominance across 23 wetland plant species. Relationships between (a) niche breadth (θ; *sensu* Zelený & Chytrý, 2019) and (b) local dominance (*log* dominance ratio) with six predictors. Bars show the signed coefficient of determination (*R* ^2^) from single‐predictor phylogenetic generalized least squares (PGLS) models, with the sign taken from the model slope: blue = positive association, red = negative association. Asterisks denote PGLS slope significance: ^m^, *P* < 0.1; *, *P* < 0.05; **, *P* < 0.01; ***, *P* < 0.001.

Competitive effect and abiotic tolerance showed positive associations with species' niche breadth and local dominance as inferred from vegetation samples (Fig. 5a,b). In both cases, competitive effect was the strongest predictor, explaining 48% of the variance in niche breadth (Fig. S11C; *P* < 0.001) and 31% in local dominance (Fig. S12C; *P* = 0.006), while abiotic tolerance explained less in both cases (Figs S11B, S12B). Niche breadth tended to be negatively correlated with RGR (Figs 5a, S11A). Local dominance was negatively associated with drought preference (Figs 5b, S12E; *R* ^2^ = 0.23, *P* = 0.022). Species occurrence showed no significant relationships with any of the six predictors (Figs S13, S14). All‐subsets PGLS model comparison supported competitive effect as the best predictor of niche breadth, with the single‐predictor model ranked first (Table S7). For local dominance, several models received comparable support (ΔAICc < 2), but competitive effect was retained across all top‐ranked models and was frequently accompanied by a positive effect of RGR, whereas support for tolerance and preference was weaker and less consistent (Table S8).

## Discussion

We provide empirical evidence for a link between experimentally quantified performance measures and species success in natural communities. Our results show that wetland plant communities exhibit a range of ecological strategies, in which competitive effect is positively associated with the operational measure of abiotic tolerance (reflecting performance consistency across hydrological regimes) but trade‐off against rapid growth. A potential limitation of our abiotic‐tolerance metric is that species performing poorly under both hydrological regimes could nevertheless appear tolerant, simply because they show little difference in biomass between treatments. However, we consider this unlikely because species with high inferred abiotic tolerance also showed strong competitive effects, which would not be expected if the pattern were driven by generally poor‐performing species.

The first three principal axes identified in our dataset broadly align with major dimensions of plant trait variation described in global syntheses (Díaz *et al*., 2016; Joswig *et al*., 2022). In particular, PC1 corresponds most closely to the global size axis; in our dataset, it is represented by traits related to persistence and stature. PC2 is related to the leaf economics spectrum, here expressed mainly as an acquisitive leaf–root morphology dimension. PC3 captures an additional physiological dimension involving stomatal conductance, δ^13^C and RMF, which is less prominently represented in global trait frameworks. Although these axes should not be understood as fully discrete syndromes, they provide a useful basis for interpreting trait covariation in our species set.

The functional traits associated with PC1 and PC3 provide a mechanistic explanation why tolerant species are at the same time competitively strong. Our results show that wetland species with higher tolerance to soil moisture gradient exhibited a distinctive combination of traits: higher competitive effects on neighbours despite lower RGR, increased LDMC, taller stature and deeper roots, higher RMF, lower δ^13^C, higher stomatal conductance, and higher stomatal fraction. At first sight, this combination appears contradictory: high LDMC and low RGR are typically associated with conservative strategies (e.g. Song *et al*., 2021), whereas high stomatal conductance and low δ^13^C indicate acquisitive water use (i.e. low water‐use efficiency; Ma *et al*., 2023). Within the conceptual framework proposed by Wang *et al*. (2022), these patterns can be reconciled. LDMC captures both structural and physiological dimensions of leaf function. Structurally, high LDMC reflects greater tissue density and investment in cell walls, leading to tougher and longer‐lived leaves and consequently lower RGR (Garnier *et al*., 2001; Poorter *et al*., 2009). However, the physiological implications of LDMC are not unidirectional. In our system, high LDMC does not appear to reflect a simple conservative syndrome (Wright *et al*., 2004), because competitively dominant species simultaneously exhibited greater height, higher LDMC, and higher leaf N concentration on a dry‐mass basis. Instead, elevated LDMC may reflect structural reinforcement and persistence associated with leaf construction, rather than reduced investment in photosynthetic tissues (Vernescu & Ryser, 2009). This interpretation is consistent with evidence that the functional consequences of leaf structural investment depend on its underlying components, as leaf thickness and tissue density can influence photosynthetic capacity in different ways (Niinemets, 1999). In wetland species, LDMC is influenced by biomechanical constraints and may covary with leaf thickness and structural support, rather than directly reflecting a conservative resource‐use strategy (Vernescu & Ryser, 2009). The fact that dominant species combined low δ^13^C, high stomatal conductance and elevated leaf nitrogen concentrations indicates that they operate at high gas‐exchange capacity (Vendramini *et al*., 2002), consistent with a strategy of maximizing carbon fluxes. Rather than reflecting weak photosynthetic capacity, this constellation suggests that successful wetland species maintain open stomata and strong biochemical uptake simultaneously. Yet, despite this high physiological throughput, their growth rates remain low, as carbon is channelled into structurally robust and persistent organs (high LDMC, large biomass, and deep roots). This aligns with the finding that LDMC can be a stronger predictor of productivity than SLA (Smart *et al*., 2017). This trade‐off between fast carbon fluxes and slow turnover reconciles the apparent paradox of acquisitive physiology with conservative growth, and explains how these species can dominate across both wet and dry environments. Even though our root trait data were limited, the observed greater MRL and biomass allocation are consistent with dimensions of the emerging root economics space (Kong *et al*., 2019; Bergmann *et al*., 2020), suggesting that belowground investment reinforces ecological breadth along the moisture gradient. Taken together, our findings indicate that the maintenance of broad ecological niches in wetlands relies on structural robustness associated with larger size, together with abiotic tolerance. This supports the view that ecological generalism can arise through multiple trait syndromes, depending on environmental context and resource availability (Niinemets, 2001; Westoby *et al*., 2002; Schellenberger Costa *et al*., 2018). Several of these traits were strongly intercorrelated, especially within the persistence–stature group. This makes it difficult to disentangle their respective contributions to tolerance and competitive effect.

Abiotic tolerance and preference showed significant phylogenetic signal, whereas competitive ability did not. Phylogenetic signal was also detected in several individual functional traits, including LDMC, stomatal fraction, RSV, δ^13^C, leaf C, and MRL, reflecting deep clade‐level differences between graminoids and eudicots. Some of these patterns are consistent with previous evidence that persistence‐related traits are often conserved across major clades (Westoby *et al*., 2002; Ackerly, 2009). By contrast, plant height, stomatal conductance, RGR, leaf N, leaf P, SLA and RMF were phylogenetically labile, in line with broader findings that photosynthetic and acquisitive traits are evolutionarily flexible and often converge repeatedly across lineages (Cavender‐Bares *et al*., 2009). Our data partly resonate with hydrological niche theory, which views preference for flooding vs drought as a deep phylogenetic trade‐off (Silvertown *et al*., 1999), shaped by adaptations, such as increased root porosity and aerenchyma formation in flood‐tolerant lineages vs deeper roots in drought‐tolerant ones (Voesenek & Bailey‐Serres, 2015). In our study, species preferences for wet vs drought treatments also showed a strong phylogenetic signal, likely reflecting conserved belowground differences associated with flooding vs drought, although our root traits provide only indirect insight into the underlying anatomical mechanisms. At the same time, however, our results extend the hydrological niche framework by showing that an additional strategy is the evolution of broad tolerance across hydrological extremes, expressed in persistence–stature traits and reinforced by physiological adjustments. We acknowledge that the detected phylogenetic signal may partly reflect the strong representation of Poales in our dataset, and that broader phylogenetic sampling could potentially modify these patterns.

Our results demonstrate that competitive effect was the dominant predictor of both local dominance and niche breadth, with abiotic tolerance being positively associated with competitive ability. This finding challenges the competition–abiotic‐tolerance trade‐off, which equates competitive strength with rapid growth in benign environments and assumes a conflict with abiotic tolerance (Grime, 1977; Carscadden *et al*., 2020). Instead, the positive association between abiotic tolerance and competitive effect indicates that competitive ability can broaden rather than constrain niche breadth, and may therefore be a stronger determinant of species occurrence in natural communities. Evidence from wetlands supports this view: species dominating the most productive sites also exhibited the broadest ranges (Bell *et al*., 2000). In this light, evolutionary constraints between abiotic tolerance and competitive ability may not be as strict as commonly assumed (Futuyma & Moreno, 1988; Martin & Ghalambor, 2023). Recent work from dry grasslands provides a parallel example, showing that abiotic‐tolerant species can also resist competition, pointing to context‐dependent synergies rather than universal antagonism (Mount *et al*., 2024). While our results still fit within the broader framework of the well‐established abiotic tolerance–growth trade‐off (Grime & Hunt, 1975), these classical studies often assumed, that competitive ability is tightly linked with rapid growth in benign environments. Our findings instead reveal that competitive ability can be positively associated with abiotic tolerance, whereas rapid growth represents an alternative strategy. Consistent with this, positive effects of RGR were retained in most of the best‐supported models predicting local dominance identified using AIC. Thus, in wetlands the key axis of niche differentiation is not abiotic tolerance vs competition, but slow‐growing, tolerant competitors vs fast‐growing colonizers (Cadotte *et al*., 2006; Reich, 2014).

From a niche‐theory perspective, it is notable that competitive effect rather than response predicted realized niche breadth. Classical theory would suggest that niche breadth should be shaped by species' tolerance to abiotic conditions together with their response to competition, as these define the limits of realized distributions (Chase & Leibold, 2003). Yet, our results show that response traits were variable and contributed little, whereas competitive effect consistently explained variation in niche breadth and local dominance (Keddy *et al*., 1994; Fraser & Miletti, 2008; Wang *et al*., 2010). This suggests that realized niches depend more on how effectively species suppress others than on how they endure competitors, shifting the focus from resistance to impact. Interestingly, species that exerted the strongest suppression on heterospecifics were also strongly self‐limited, suggesting that the same persistence–stature traits drive density dependence within populations as well as across species. By contrast, drought‐adapted wetland species exhibited particularly weak interspecific response, a pattern consistent with theory that variation in response traits reduces mutual limitation and thereby promotes local coexistence (Kraft *et al*., 2015; Cadotte, 2017). Such mechanisms may help maintain species diversity in wetlands, despite the well‐known tendency of these ecosystems to be dominated by a few species.

Our results show that wetland plant dominance is driven not by rapid growth, but by persistence–stature traits that couple abiotic tolerance with strong competitive effects. This mechanism refines classical trade‐off models by revealing that tolerance and competitiveness can reinforce each other rather than conflict. Such trait profiles may help predict shifts in community composition under global change: species lacking them may be especially vulnerable to more frequent and intense environmental extremes, whereas abiotic‐tolerant dominants may expand their dominance, potentially reducing diversity. Whether similar trait combinations underpin dominance in other ecosystems is not well known, but parallels with tall dominants in grasslands and other productive habitats suggest broader relevance.

## Competing interests

None declared.

## Author contributions

J Douda and J Doudova conceived the study, developed the methodology, collected the data, analysed the data, and wrote the manuscript. PK contributed to the methodology and data management. PK, JH, AH, LE and AH collected the data and maintained the experiment. JK performed the isotope analyses. J Douda and J Doudova contributed equally to this work. All authors contributed to the writing of the manuscript.

## Disclaimer

The New Phytologist Foundation remains neutral with regard to jurisdictional claims in maps and in any institutional affiliations.

## Supporting information


**Fig. S1** Phylogeny–trait bubble diagram.
**Fig. S2** Daily precipitation totals and mean daily air temperatures during the 2023 experimental season, measured at the nearest meteorological station (Ruzyně; 50°06′1.16″N, 14°15′19.97″E).
**Fig. S3** Experimental design and setup.
**Fig. S4** Posterior distributions of PGLS slopes for Fig. 1.
**Fig. S5** Relationships with relative growth rate estimated from plant height.
**Fig. S6** Relationships between measures of competitive ability in studied species.
**Fig. S7** Posterior distributions of PGLS slopes for Fig. S6.
**Fig. S8** Posterior distributions of PGLS slopes for Fig. 2.
**Fig. S9** Variance of PCA axes based on 18 traits across 23 species.
**Fig. S10** Absolute loadings of the first four trait PCA axes.
**Fig. S11** Niche breadth and metrics of abiotic tolerance and competitive ability.
**Fig. S12** Local dominance and metrics of abiotic tolerance and competitive ability.
**Fig. S13** Species occurrence and metrics of abiotic tolerance and competitive ability.
**Fig. S14** Predicted species occurrence based on ecological strategies.
**Methods S1** Seed stratification; seed germination and seedling cultivation; transplantation and replacement.
**Methods S2** Hydrological regimes.
**Methods S3** Statistical framework for biomass prediction models.
**Methods S4** Functional traits approach.
**Methods S5** Intraspecific competition experiment.
**Methods S6** Quantifying realized niche breadth from co‐occurrence data.
**Table S1** Numbers of individuals cultivated and transplanted within each treatment.
**Table S2** Results of species‐specific mixed models predicting aboveground biomass from morphological traits.
**Table S3** Overview of functional traits measured in the study.
**Table S4** PGLS model comparison for competitive effect, interspecific response, and intraspecific response.
**Table S5** Pairwise Pearson correlations among functional traits.
**Table S6** Pairwise phylogenetically corrected Pearson correlations among functional traits.
**Table S7** Model comparison of PGLS models explaining species niche breadth.
**Table S8** Model comparison of PGLS models explaining local dominance.
Please note: Wiley is not responsible for the content or functionality of any Supporting Information supplied by the authors. Any queries (other than missing material) should be directed to the *New Phytologist* Central Office.

## Data Availability

The data and R code required to fully reproduce all analyses are available on Zenodo (doi: 10.5281/zenodo.17647705).
