# Supplementary material for: A trait‐based integration of competition and abiotic tolerance underlies plant success
Source: New Phytol. 2026 Jul 23;252(1):87–99. doi: 10.1111/nph.71465 (PMC13539952; doi:10.1111/nph.71465)
Supplement: Supplementary file 1 [file NPH-252-87-s001.pdf]

New Phytologist Supporting Information

Article title: A trait-based integration of competition and abiotic tolerance underlies plant success

Authors: Jan Douda, Jana Doudová, Přemysl Král, Jan Häusler, Anežka Holeštová, Eusebio Leandro, Jiří Květoň, Alena Havrdová

Article acceptance date: 04 July 2026

The following Supporting Information is available for this article:

Supplementary Methods

S1. Seed stratification; Seed germination and seedling cultivation; Transplantation and replacement

S2. Hydrological regimes

S3. Statistical framework for biomass prediction models

S4. Functional traits approach

S5. Intraspecific competition experiment

S6. Quantifying realized niche breadth from co-occurrence data

Supplementary Figures

Fig. S1. Phylogeny–trait bubble diagram

Fig. S2. Daily precipitation totals and mean daily air temperatures during the 2023 experimental season, measured at the nearest meteorological station (Ruzyně; 50°06′1.16″ N, 14°15′19.97″ E).

Fig. S3. Experimental design and setup

Fig. S4. Posterior distributions of PGLS slopes for Fig. 1

Fig. S5. Relationships with relative growth rate estimated from plant height

Fig. S6. Relationships between measures of competitive ability in studied species

Fig. S7. Posterior distributions of PGLS slopes for Fig. S6

Fig. S8. Posterior distributions of PGLS slopes for Fig. 2

Fig. S9. Variance of PCA axes based on 18 traits across 23 species

Fig. S10. Absolute loadings of the first four trait PCA axes

Fig. S11. Niche breadth and metrics of abiotic tolerance and competitive ability

Fig. S12. Local dominance and metrics of abiotic tolerance and competitive ability

Fig. S13. Species occurrence and metrics of abiotic tolerance and competitive ability

Fig. S14. Predicted species occurrence based on ecological strategies

Supplementary Tables

Table S1. Numbers of individuals cultivated and transplanted within each treatment

Table S2. Results of species-specific mixed models predicting above-ground biomass from morphological traits

Table S3. Overview of functional traits measured in the study

Table S4. PGLS model comparison for competitive effect, interspecific response, and intraspecific response

Table S5. Pairwise Pearson correlations among functional traits

Table S6. Pairwise phylogenetically corrected Pearson correlations among functional traits

Table S7. Model comparison of PGLS models explaining species niche breadth

Table S8. Model comparison of PGLS models explaining local dominance

Supplementary Methods S1

Species selection

Species selection was based on the classification of wetland vegetation presented in *Vegetation of the Czech Republic* (Chytrý 2011). We focused on two vegetation classes representing common wetland habitats in the Czech Republic: the herbaceous littoral wetland class *Phragmito-Magno-Caricetea* Klika in Klika et Novák 1941 and the shrub wetland class *Alnetea glutinosae* Br.-Bl. et Tüxen ex Westhoff et al. 1946. Candidate species were selected from the synoptic tables of these classes and included either diagnostic species of the constituent associations, identified on the basis of fidelity expressed by a significant phi coefficient (Chytrý 2011), or species occurring with a frequency higher than 40% in the respective associations. This procedure ensured that the selected species had a strong phytosociological affinity to wetland communities.

Seed stratification

In February 2023, seeds underwent stratification in Petri dishes at 4°C under moist, dark conditions for one month to break dormancy, using a seed germinator (Hulík and Douda 2017). Concurrently, natural stratification was initiated for each species in two 90 L plastic containers (360 mm height, 660 mm diameter; IKO90 CONTAINER, KAXL, Bílovec, Czech Republic) filled with a soil and sand mixture (2:1). These conditions provided more variable germination environments that could facilitate the establishment of some species.

Seed germination and seedling cultivation

After one month of stratification, seeds in germination boxes were exposed to a 16/8 hour (day/night) light regime with a temperature cycle of 22/10°C to induce germination. Germination occurred progressively during March and April. Germinated seedlings were transferred into seedling trays filled with soil and grown in a greenhouse under ambient temperatures. Before transplantation, these seedlings were acclimatized to outdoor conditions by being gradually moved outside the greenhouse. Seeds stratified in the 90 L containers germinated naturally during March and April. These containers were regularly watered, and seedlings were allowed to grow in situ until transplantation.

Transplantation, replacement and fertilization

At 10–12 May, seedlings were transplanted into experimental tubes positioned in tanks. Priority was given to seedlings cultivated in the greenhouse to ensure uniform size, with seedlings from

the 90 L containers used as a supplement when greenhouse-grown seedlings were insufficient. Over the following four weeks, any plants that died, likely due to transplantation stress, were replaced with seedlings of the same species or, if unavailable, a different species. This ensured that all dead plants were replaced to maintain two species in each competitive pair and to increase the number of replicates for some species in both the wet and dry treatments (see Table S1). During the period of seedling transplantation (10 May–9 June; Figure S2), watering was applied only moderately to maximize plant establishment before the fully irrigated regime was implemented. Plants were fertilized with an organo-mineral fertilizer (poultry manure–based) containing 40% dry matter, 8.5% total N, 7.8% P_2O_5, and 9.5% K_2O. The fertilizer contained at least 55% combustible substances and had a pH of 4.5–6.5. Fertilization was applied three times during the growing season (20 June, 29 June, and 10 August).

Supplementary Methods S2

Hydrological regimes

To allow meaningful interspecific comparisons, we defined drought as a standardized external condition rather than as a species-specific physiological threshold (McDowell et al. 2008; Gilbert and Medina 2016). It aligns with efforts to simulate realistic environmental scenarios while capturing species-specific physiological responses to externally imposed water limitation. At the beginning of the experiment (9 June), all tanks were fully waterlogged to ensure uniform soil saturation. The high-water and competition treatments were maintained at the same water level throughout the growing season. They were maintained by keeping the water table at 5.9 ± 1.4 cm below the soil surface. This corresponded to an average volumetric soil water content of 32.2 ± 2.2%, with values close to 36% representing full irrigation capacity (40–60% for most soils; moisture measured using TDR Trime Pico 64 sensors, Imko GmbH). In the drought treatment, water level reduction was carried out in two steps (Figure S2). After 14 days of full irrigation (23 June), the water table was lowered to 10 cm below the soil surface, resulting in a volumetric soil water content of 28.8 ± 1.8%. After another 14 days (6 July), it was further reduced to 20 cm and subsequently maintained at 32.3 ± 1.7 cm below the soil surface for the rest of the season. This final stage corresponded to a mean volumetric soil water content of 13.1 ± 4.0%. Detailed arrangement of treatments in the garden and tank dimensions are shown in Figure S3.

Supplementary Methods S3

Statistical framework for biomass prediction models

We used sets of non-destructive morphological traits (i.e. number of ramets, plant height, leaf length, number of branches, and number of leaves) as predictors to fit species-specific models of above-ground biomass harvested at the end of the growing season. Generalized linear models (GLMs) or generalized linear mixed models (GLMMs) were fitted depending on model performance. All models assumed a Gamma error distribution with a log link function, which is appropriate for strictly positive, right-skewed biomass data. When inclusion of tank identity as a random intercept improved model fit (assessed via AIC), we used GLMMs implemented with the *glmer* function in the lme4 package. For some species, random effects did not improve the model, and simpler GLMs (glm function) were used instead. For each species, the best-performing model was selected based on AIC (Table S2). The resulting model equations (with trait coefficients) were then used to predict above-ground biomass at all seven time points for each individual. These biomass predictions formed the input for modeling species growth trajectories. For each plant, we interpolated the time series of biomass values using generalized additive models (GAMs; gam function from the mgcv package), again assuming a Gamma distribution and a log link to accommodate the skewed nature of biomass data. Smooth terms were fit with a maximum of five knots to avoid overfitting. From the smoothed biomass trajectories, we calculated daily absolute growth rate (AGR) as the first derivative (i.e., daily change in biomass), and relative growth rate (RGR) as the AGR divided by the biomass at that time point. This yielded time-resolved estimates of species-specific growth performance across treatments.

Supplementary Methods S4

Functional trait approach

To characterize functional strategies of the studied species, we measured a suite of morphological, physiological, and chemical traits following standardized protocols within the applied experimental treatments. **Leaf dry matter content** (LDMC) was calculated as the ratio of oven-dry leaf mass to fresh mass measured directly after harvest. **Specific leaf area** (SLA) was calculated as the ratio of leaf area to dry mass, with leaf area measured using a leaf area scanner. To quantify nutrient status and physiological strategies of the species, we measured carbon (C), nitrogen (N), phosphorus (P) content and carbon isotope composition ($\delta^{13}C$) in

mature, fully expanded leaves. After collection, leaf samples were dried at 60 °C for 48 hours. **Total leaf carbon** and **nitrogen concentrations** (% dry mass) were measured using dry combustion in an elemental analyzer (Flash 2000, Thermo Scientific). Finely ground plant material (< 0.1 mm) was weighed into tin capsules (5–10 mg depending on expected content) and combusted at 1000 °C in the presence of chromium oxide as a catalyst and pure oxygen as the carrier gas. The resulting gases were passed through a reduction column (800 °C, copper), dried, separated chromatographically, and detected by a thermal conductivity detector. Data were evaluated using the EagerSmart software (Thermo Fisher Scientific). **Leaf phosphorus concentration** (mg kg^{-1} dry mass) was determined colorimetrically after microwave-assisted acid digestion (Milestone Standard 1200 Mega; ~0.5 g plant material with 4 ml HNO_3 and 2 ml 30% H_2O_2). After filtration, orthophosphate was quantified using the molybdenum blue method (Olsen protocol) with sulfuric acid, ascorbic acid, and potassium antimony tartrate. Absorbance was measured at 630 nm using a UV-vis spectrophotometer (UV-400, Unicam). **Stable carbon isotope composition** ($\delta^{13}C$) was measured in a subset of dried and finely ground leaf samples. Approximately 2.0–2.7 mg of material was weighed into tin capsules and analyzed using continuous-flow isotope ratio mass spectrometry (IRMS) (Delta plus XL, Thermo Finnigan), coupled to an elemental analyzer (Flash 2000, Thermo Fisher Scientific). The $\delta^{13}C$ values are expressed in per mil (‰) relative to the Vienna Pee Dee Belemnite (VPDB) standard and were used as an integrative proxy for intrinsic water-use efficiency (Farquhar et al. 1989). Instrument calibration was performed using international reference materials and laboratory working standards with known isotopic composition, and analytical precision was typically better than ±0.06‰ (sample standard deviation). **Plant height** was measured as the distance from the soil surface to the highest photosynthetically active leaf on the tallest ramet. **Stomatal conductance** was measured using a METER SC-1 Leaf Porometer, which estimates leaf conductance to water vapor (mmol m^{-2} s^{-1}) by quantifying vapor diffusion through stomata. During measurement, the sensor head was briefly opened and clamped around a representative portion of the leaf (fully covering the sensor window), and conductance was recorded after 30 seconds. Excess moisture was removed from the desiccant between readings. Both leaf surfaces were measured when feasible. For species with cylindrical or atypically shaped leaves (e.g., *Juncus effusus*), stomatal conductance could not be reliably measured. Measurements were conducted between July 11 and August 23, 2023, during two daily time windows (11:00–12:00 and 15:00–16:00). Each plant was measured once during the experiment, with both morning and afternoon measurements taken on the same day. Stomatal conductance was calculated as the sum of adaxial and abaxial leaf conductance measured in

both morning and afternoon for each plant. Specifically, conductance values from both leaf surfaces were summed separately for the morning (early) and afternoon (late) measurements. Then, a mean daily conductance was computed for each individual by averaging the early and late values. **Stomatal pore area fraction** was calculated as the product of stomatal density (d) and stomatal size (s). Stomatal density (number of stomata per mm^2) was estimated from two replicate counts per surface, based on the number of stomata within a circular microscope field ($r = 0.2$ mm; area $S = \pi r^2$). Stomatal pore size (μm^2) was calculated as the area of an ellipse from stomatal width (x) and length (y) ($x \times y \times \pi / 4$), with up to six stomata measured per sample. Mean values of stomatal pore size were used per surface, and stomatal pore area fraction was computed as $d \times s \times 10^{-4}$, representing the proportion of epidermal surface occupied by stomatal pores. For each individual, total stomatal pore area fraction was calculated as the average of values from the adaxial (upper) and abaxial (lower) leaf surfaces. To assess belowground traits, we carefully excavated entire plants at the end of the experiment, removing soil by hand and rinsing roots under running water. For each plant, we measured **maximum root length** (MRL) as the distance from the root base to the tip of the longest root. **Root specific volume** (RSV, mL mg^{-1}) was used as an indirect proxy for variation in root structure potentially associated with porosity and aerenchyma development. RSV was calculated as the fresh volume (mL) of an approximately 10 cm root segment divided by its rehydrated fresh mass (mg). Root volume was determined by water displacement in a graduated cylinder. Higher RSV values indicate greater root porosity, as a larger volume relative to mass reflects a higher proportion of air spaces within the root tissue. We harvested above- and belowground biomass separately and dried all material at 60 °C for 48 h to constant mass. **Root mass fraction** (RMF) was calculated as belowground biomass divided by total biomass. Biomass, stomatal conductance, plant height, maximum root length, and root porosity were measured for all individuals across all treatments. For the remaining traits, which are more analytically demanding and costly (e.g. LDMC, SLA, nutrient content, and carbon isotope deviation), we measured all replicates under the drought and flooding treatments, and a random subset of six individuals under the competition treatment – ensuring equal replication across treatments.

Supplementary Methods S5

Intraspecific competition experiment

This experiment aimed to assess inter- and intraspecific competition, but with only two heterospecific competitors per focal species, it does not allow precise estimation of interspecific effects and responses. However, it enables evaluation of intraspecific competition. Each target species was grown in monoculture at three densities (1, 3, and 6 individuals per container) and in mixtures with heterospecific competitor using matching densities (4, 6, 7, 9, 12), in containers measuring 30 × 30 × 30 cm filled with the same soil–sand mixture as in the main experiment. Above-ground biomass was estimated non-destructively using trait-based predictive models developed from the main dataset. To estimate intraspecific competition strength, we fitted Bayesian regression models where the biomass of a target plant was modelled as a function of intraspecific and heterospecific density, both treated as continuous covariates. Random effects for tank were initially considered but excluded due to negligible variance explained. The posterior distribution of the intraspecific density coefficient was extracted for each species, summarized by the posterior median, and standardized using a z-score transformation.

Supplementary Methods S6

Quantifying realized niche breadth from co-occurrence data

We extracted all plots containing at least one of the 23 focal wetland species from the Czech National Phytosociological Database (Chytrý and Rafajová, 2003) and retained only herb-layer data (39,831 plots). To avoid spatial bias and redundancy due to dense sampling in certain areas, we applied a spatial thinning procedure: for each species, only one plot was retained per ~1 km^2 grid cell, based on random selection within each cell (19,420 plots). The number of plots retained for each species after spatial stratification, together with corresponding values of θ and local dominance, are provided in Table A Species' realized niche breadth was quantified using the co-occurrence-based θ index (Fridley et al. 2007; Zelený and Chytrý 2019), which measures the compositional heterogeneity of vegetation plots containing the focal species. The index is derived from multiplicative beta diversity, $\beta\_W = \gamma / \alpha$, where γ is the total species richness across *n* plots and α is the mean richness per plot. We used Sørensen dissimilarity to measure compositional distances and removed outlier plots following the distance-based algorithm of McCune and Mefford (1999), excluding plots with mean dissimilarity > mean + 2 SD. To standardize for different sample sizes, we used rarefaction to $n = 10$ plots and calculated θ as the mean β_W from 100 random subsamples (function calculate.theta(), R package theta). Higher θ values correspond to greater compositional heterogeneity and thus broader ecological niche.

Table A. Final species occurrence frequencies after spatial stratification, used for the calculation of θ (index of niche breadth) and species local dominance.

Species	Occur. freq.	Theta (θ)	Dominance
Carex acuta	1989	5.95	23.49
Carex acutiformis	686	6.15	26.79
Carex elata	230	5.62	23.48
Carex elongata	366	5.14	6.92
Carex paniculata	253	5.75	27.87
Carex pseudocyperus	209	6.02	17.27
Carex remota	1080	5.22	7.54
Carex riparia	450	6.30	31.02
Cirsium palustre	3891	5.27	2.99
Deschampsia cespitosa	6368	6.13	5.92
Epilobium hirsutum	681	6.52	4.39
Glyceria fluitans	1788	6.71	15.08
Glyceria maxima	1379	6.43	26.43
Iris pseudacorus	972	6.21	10.89
Juncus effusus	4753	5.99	6.93
Lysimachia vulgaris	4382	6.18	4.57
Lythrum salicaria	2665	6.56	2.65
Myosotis palustris agg.	5057	5.64	3.44
Phalaris arundinacea	3390	6.74	14.45
Poa trivialis	5640	6.03	5.09
Rumex conglomeratus	185	6.47	2.87
Rumex sanguineus	264	5.31	2.07
Scirpus sylvaticus	3441	5.22	17.68

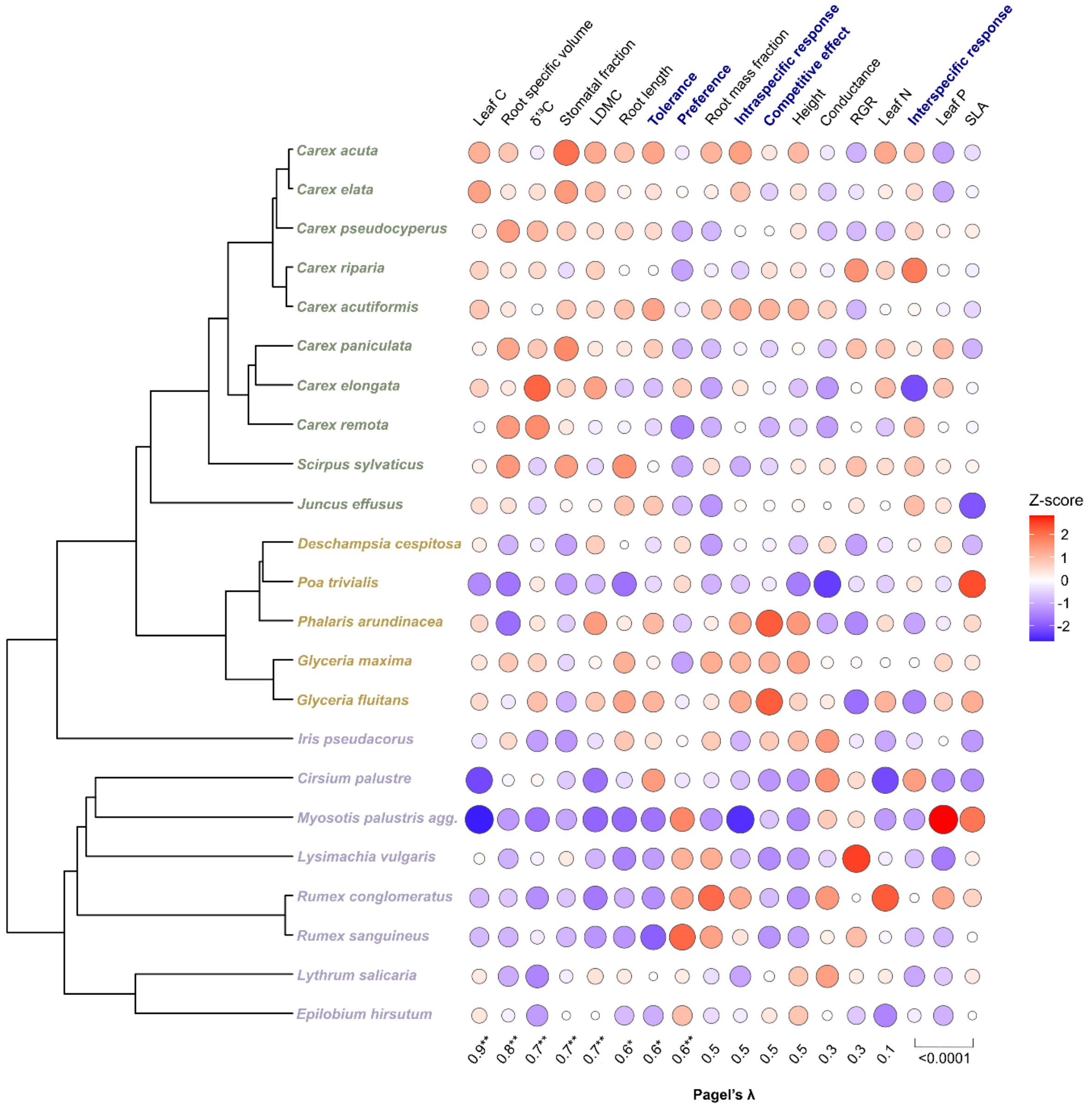

Fig. S1. Phylogeny–trait bubble diagram. Phylogeny of 23 common wetland plant species with associated functional traits, with species names colour-coded by growth form (sedge-like = grey, grasses = ochre, forbs = lilac). Circle colour and size encode the standardized trait value (Z-score): blue = below the cross-species mean, white = mean, red = above mean. Trait columns are ordered by decreasing phylogenetic signal (Pagel's λ, shown below each column). Asterisks denote significance of the λ test: * $p < 0.05$, ** $p < 0.01$, *** $p < 0.001$.

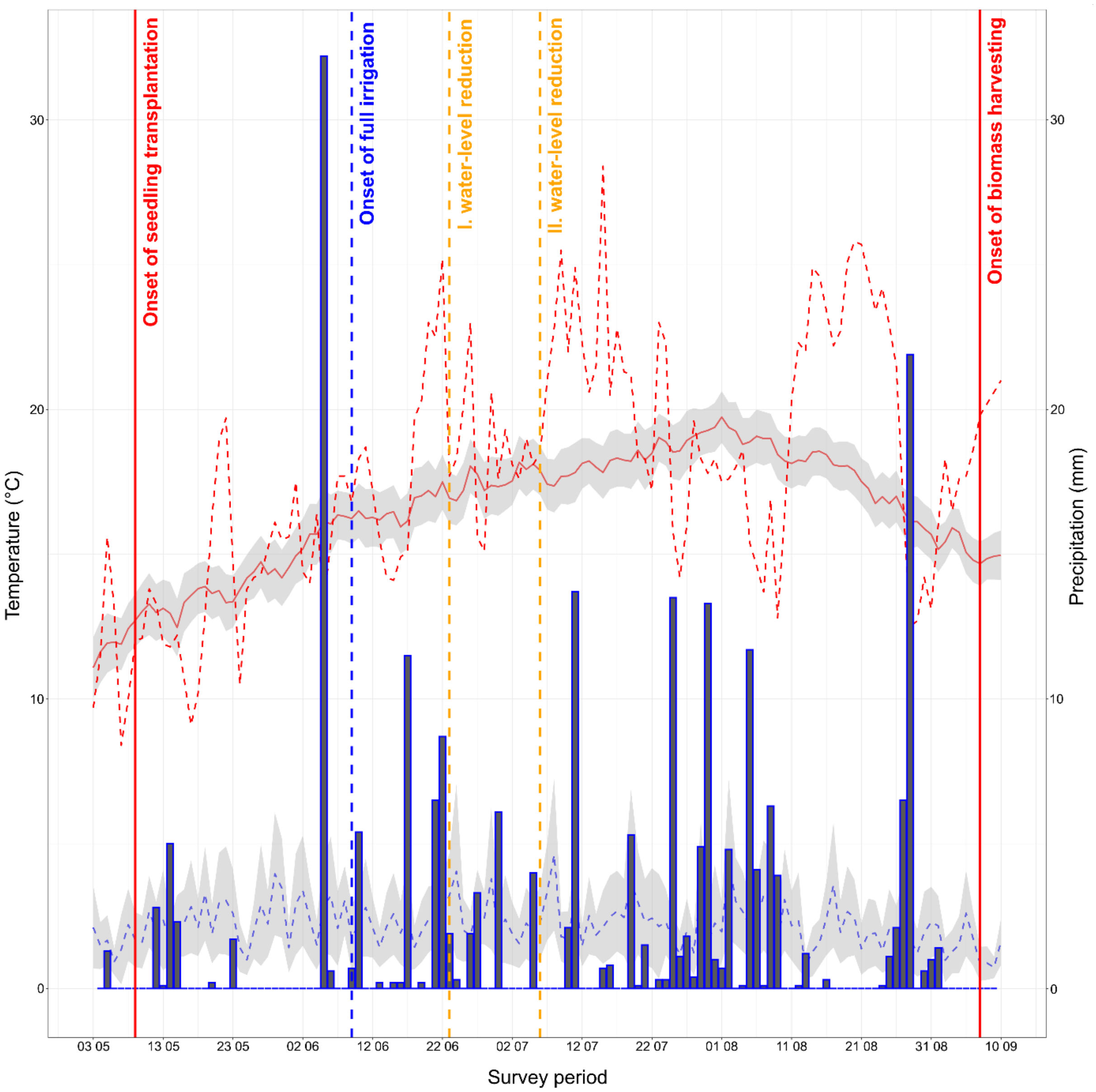

Fig. S2. Daily precipitation totals (blue bars) and mean daily air temperatures (dashed red line) during the 2023 experimental season, measured at the nearest meteorological station (Ruzyně; 50°06′1.16″ N, 14°15′19.97″ E). The dashed blue line represents the long-term mean daily precipitation for the reference period (1961–2023), while the solid red line indicates the long-term mean daily air temperature for the same period. Vertical lines mark key experimental milestones: solid red lines indicate the beginning and end of the experiment, the dashed blue line marks the initial full irrigation of all experimental tanks, and dashed golden lines denote the successive steps of water-level reduction in the drought treatment.

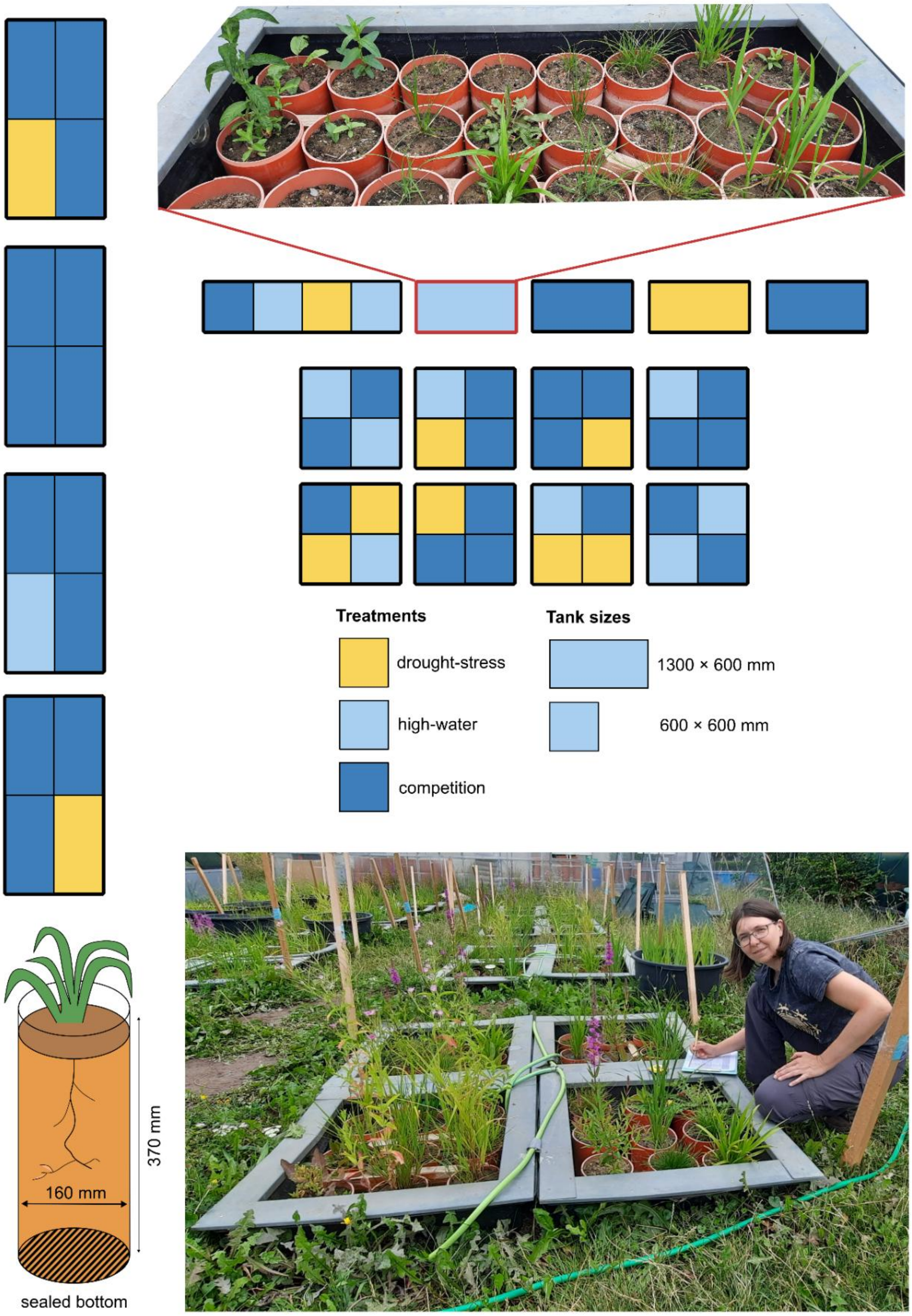

Fig. S3. Experimental design and setup. The top photo shows a large tank (1300 × 600 mm) with mid-June plants, and the bottom photo shows several smaller tanks with early-August plants (600 × 600 mm). A schematic of a single tube, in which individuals were grown either singly or in pairs in the competition experiment, is shown in the lower left corner.

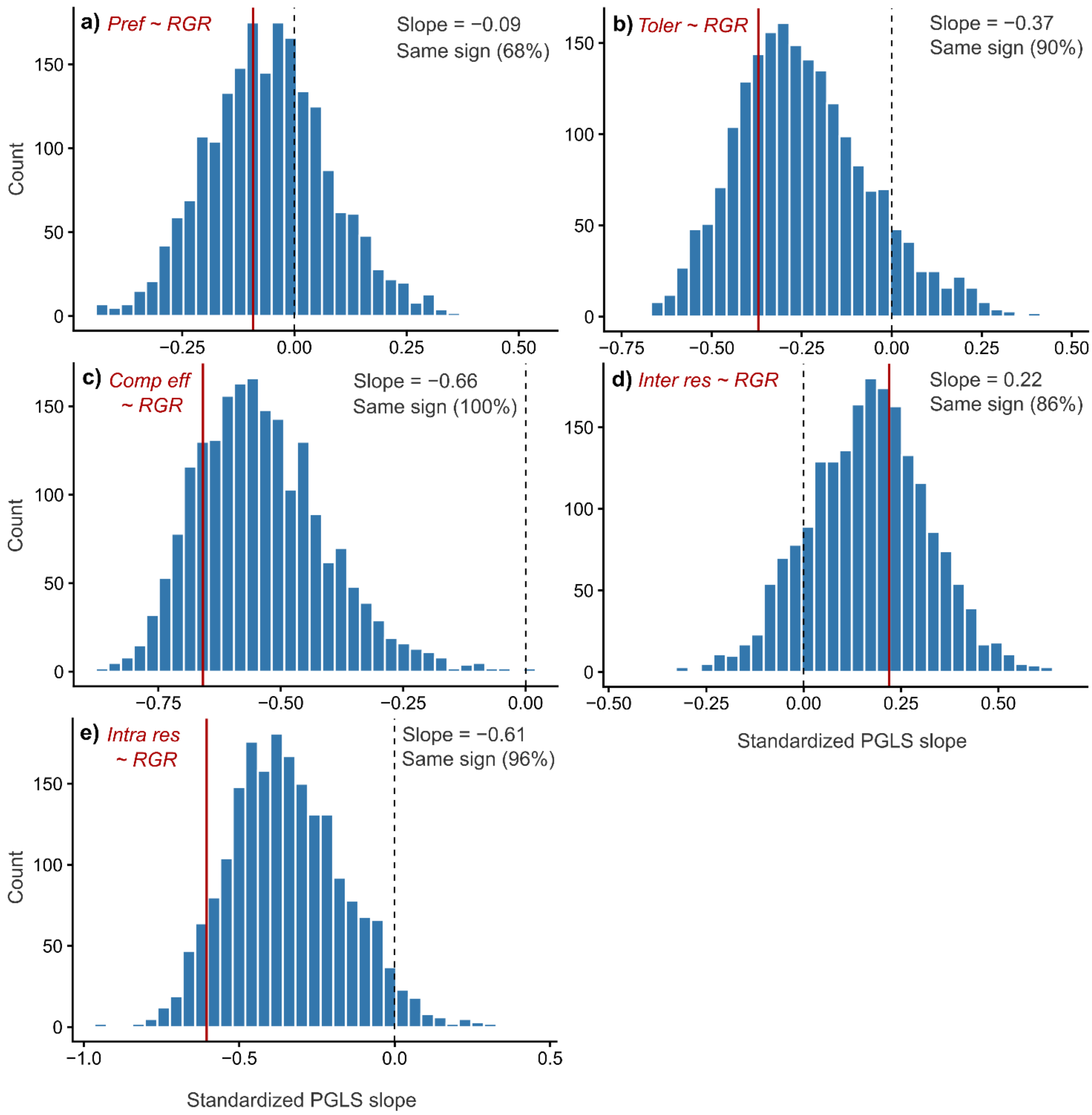

Fig. S4. Histograms show the distribution of 2,000 posterior-resampled standardized PGLS slopes for the relationships shown in the corresponding panels (a–e) of Fig. 1. The x-axis represents the standardized PGLS slope. The solid red vertical line marks the slope estimated from the median-based model. The subtitle of each panel gives the median-based slope and the percentage of posterior refits with the same slope sign. Values closer to 100% indicate stronger support for a stable slope direction, whereas values closer to 50% indicate little directional support under posterior uncertainty.

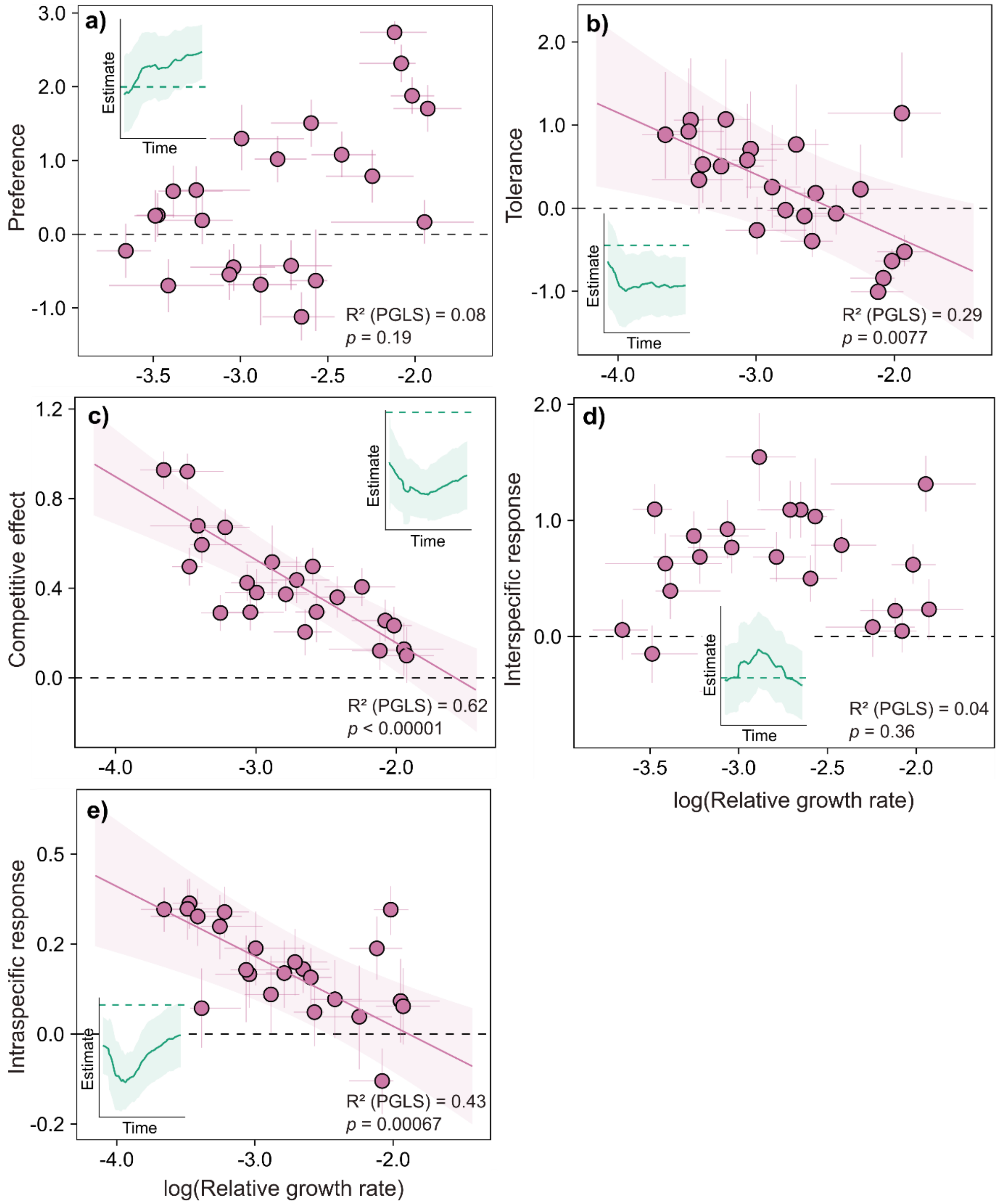

Fig. S5. Relationships between relative growth rate (estimated based on plant height) and measures of abiotic preference (**a**), abiotic tolerance (**b**), competitive effect (**c**), interspecific response (**d**), and intraspecific response (**e**) in 23 wetland plant species. Points represent species-level median estimates; error bars show 50% confidence intervals. Lines are phylogenetic generalised least-squares (PGLS) fits with its 95% confidence band. The bottom-left inset depicts the seasonal trajectory of the standardised PGLS slope (green line) with its 95% confidence band and the dashed green horizontal line indicates null slope.

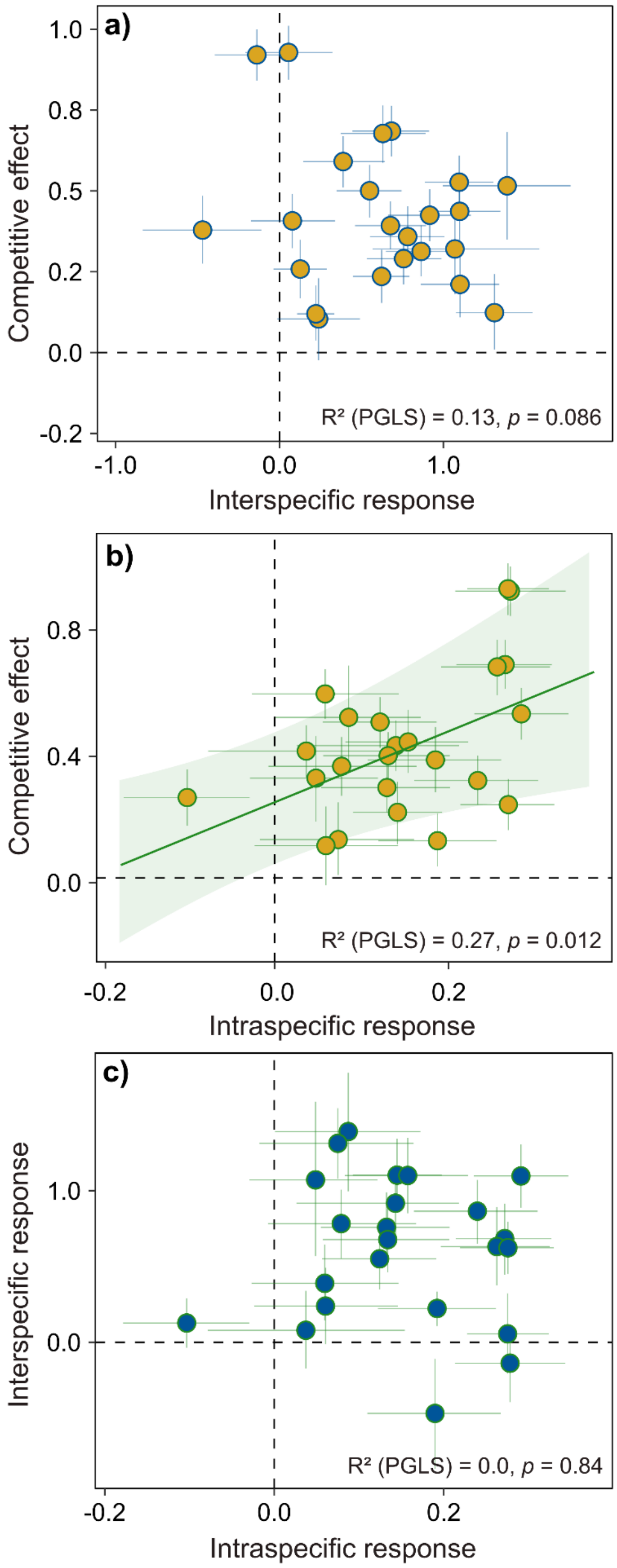

Fig. S6. Relationships between measures of competitive ability in 23 wetland plant species. Points are species-level estimates; error bars show 50% confidence intervals. Lines are phylogenetic generalised least-squares (PGLS) fits with its 95% confidence band.

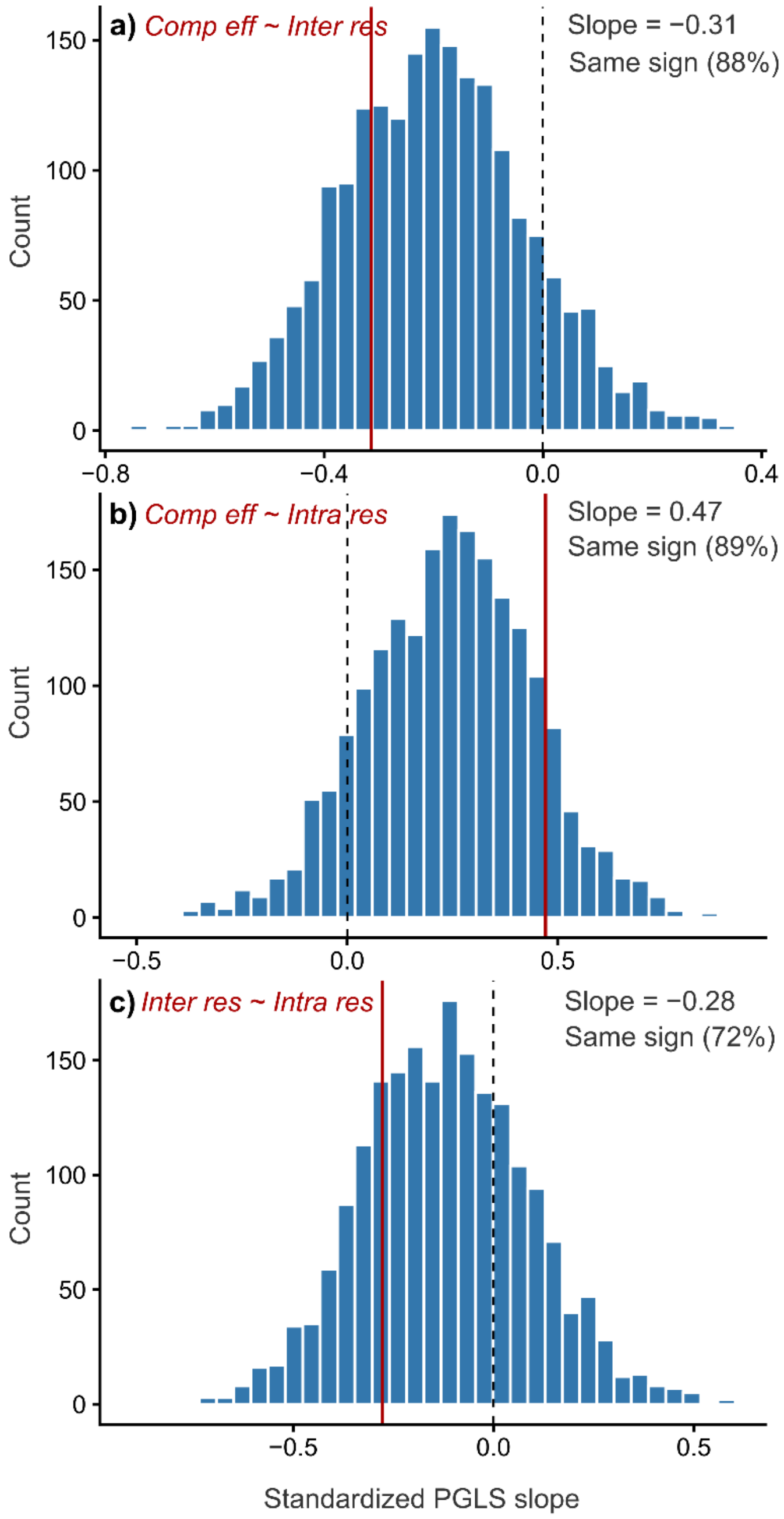

Fig. S7. Histograms show the distribution of 2,000 posterior-resampled standardized PGLS slopes for the relationships shown in the corresponding panels (a–c) of Fig. S6. The x-axis represents the standardized PGLS slope. The solid red vertical line marks the slope estimated from the median-based model. The subtitle of each panel gives the median-based slope and the percentage of posterior refits with the same slope sign. Values closer to 100% indicate stronger support for a stable slope direction, whereas values closer to 50% indicate little directional support under posterior uncertainty.

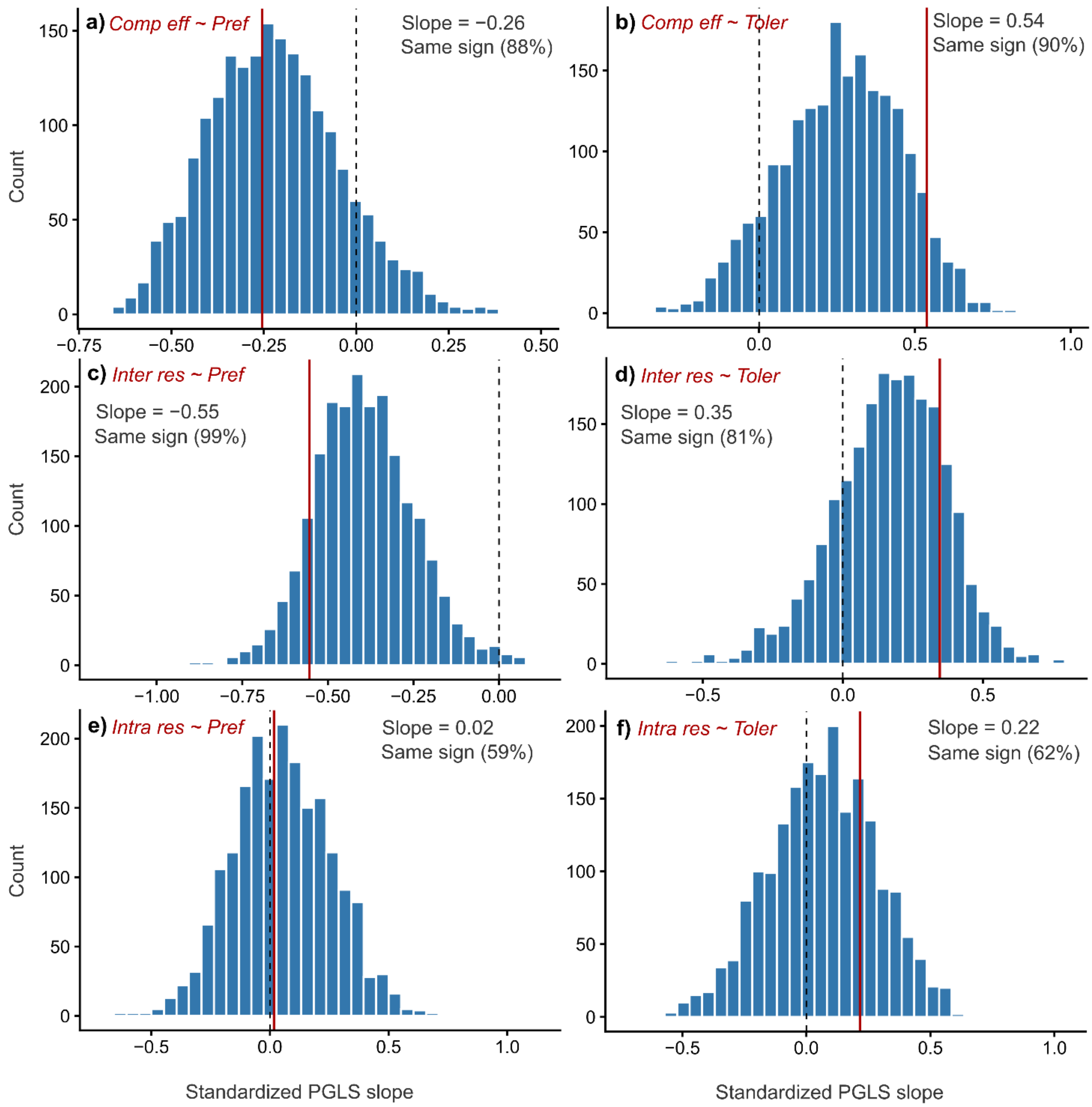

Fig. S8. Histograms show the distribution of 2,000 posterior-resampled standardized PGLS slopes for the relationships shown in the corresponding panels (a–f) of Fig. 2. The x-axis represents the standardized PGLS slope. The solid red vertical line marks the slope estimated from the median-based model. The subtitle of each panel gives the median-based slope and the percentage of posterior refits with the same slope sign. Values closer to 100% indicate stronger support for a stable slope direction, whereas values closer to 50% indicate little directional support under posterior uncertainty.

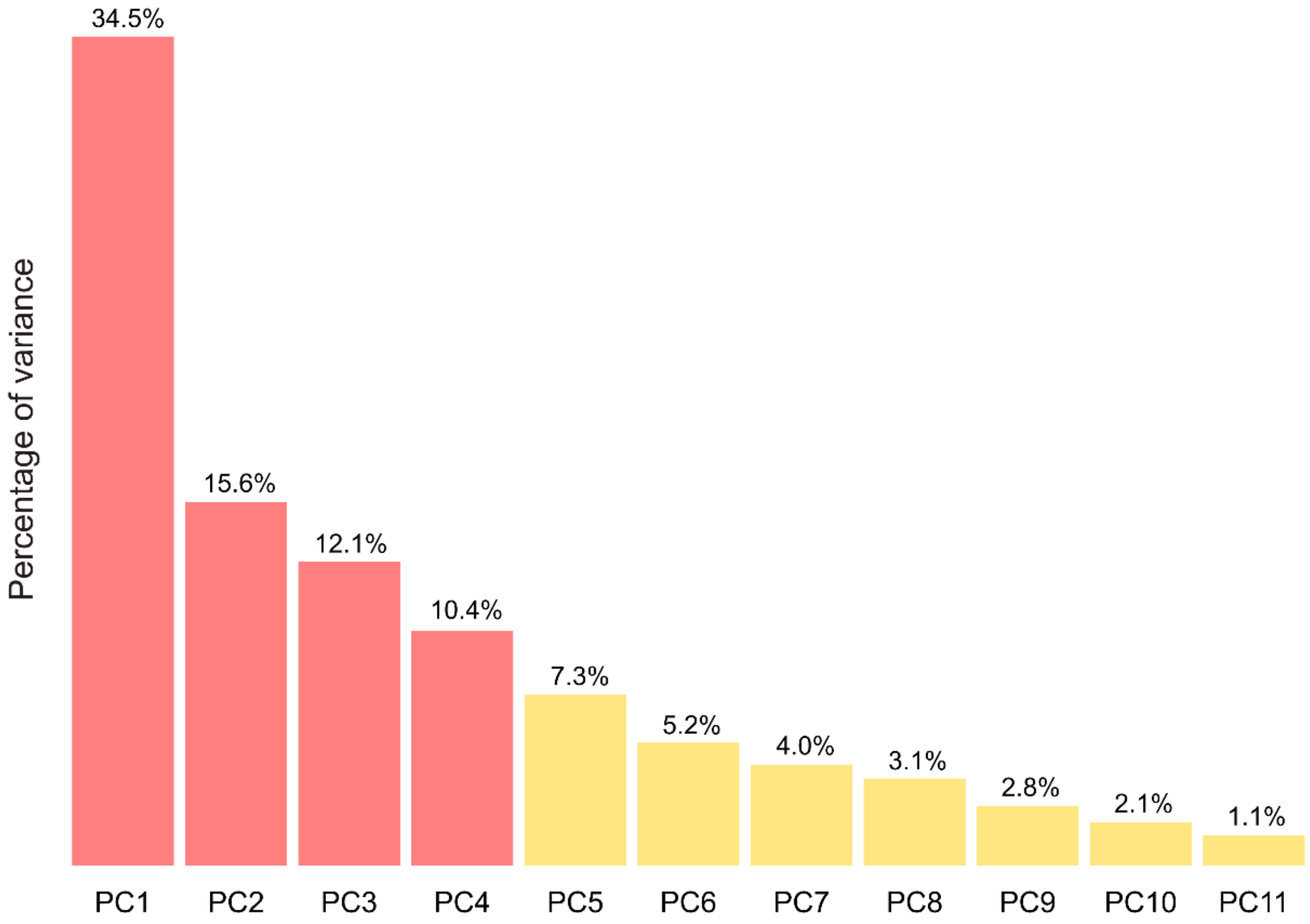

Fig. S9. Variance of the trait PCA axes (18 traits, n = 23 species). The red bars indicate more than 10% of the explained variance.

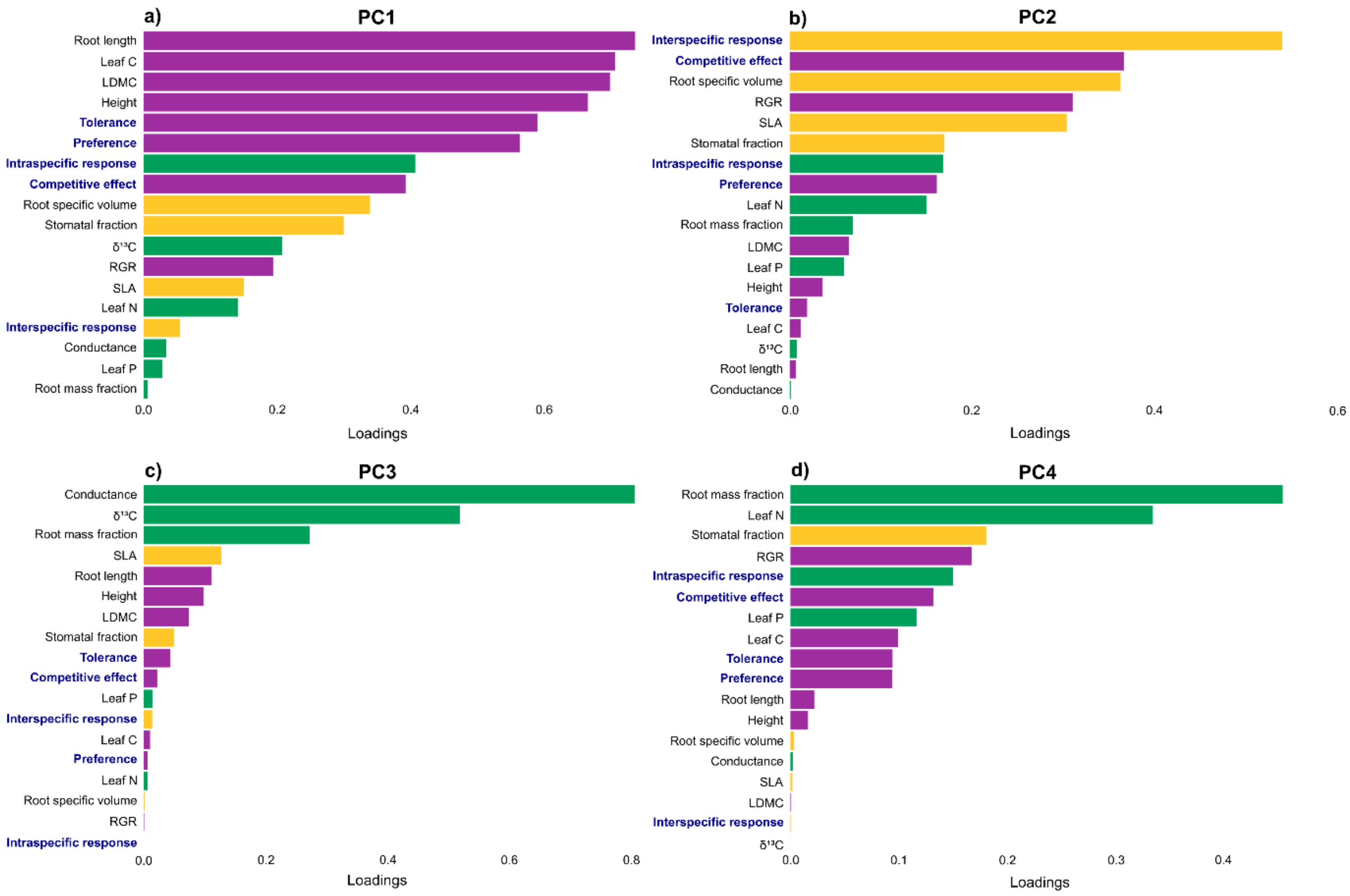

Fig. S10. Absolute loadings of the first four trait PCA axes, trait bars coloured according to three clusters (violet = persistence–stature traits, yellow = leaf–root acquisitive morphology, green = physiological–acquisitive traits).

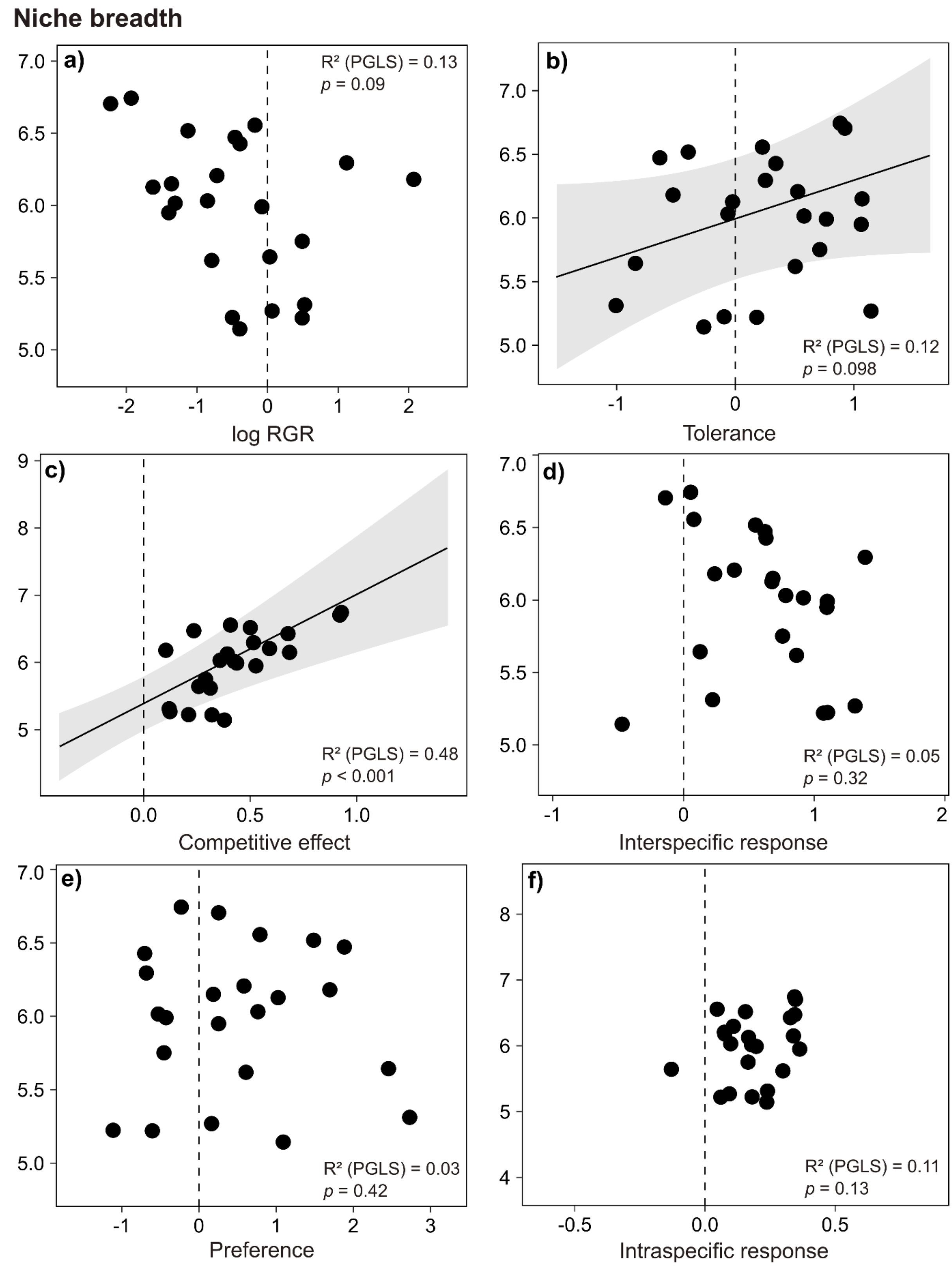

Fig. S11. Relationships between niche breadth and metrics of abiotic tolerance and competitive ability in 23 wetland plant species. Lines are phylogenetic generalised least-squares (PGLS) fits with 95% confidence bands.

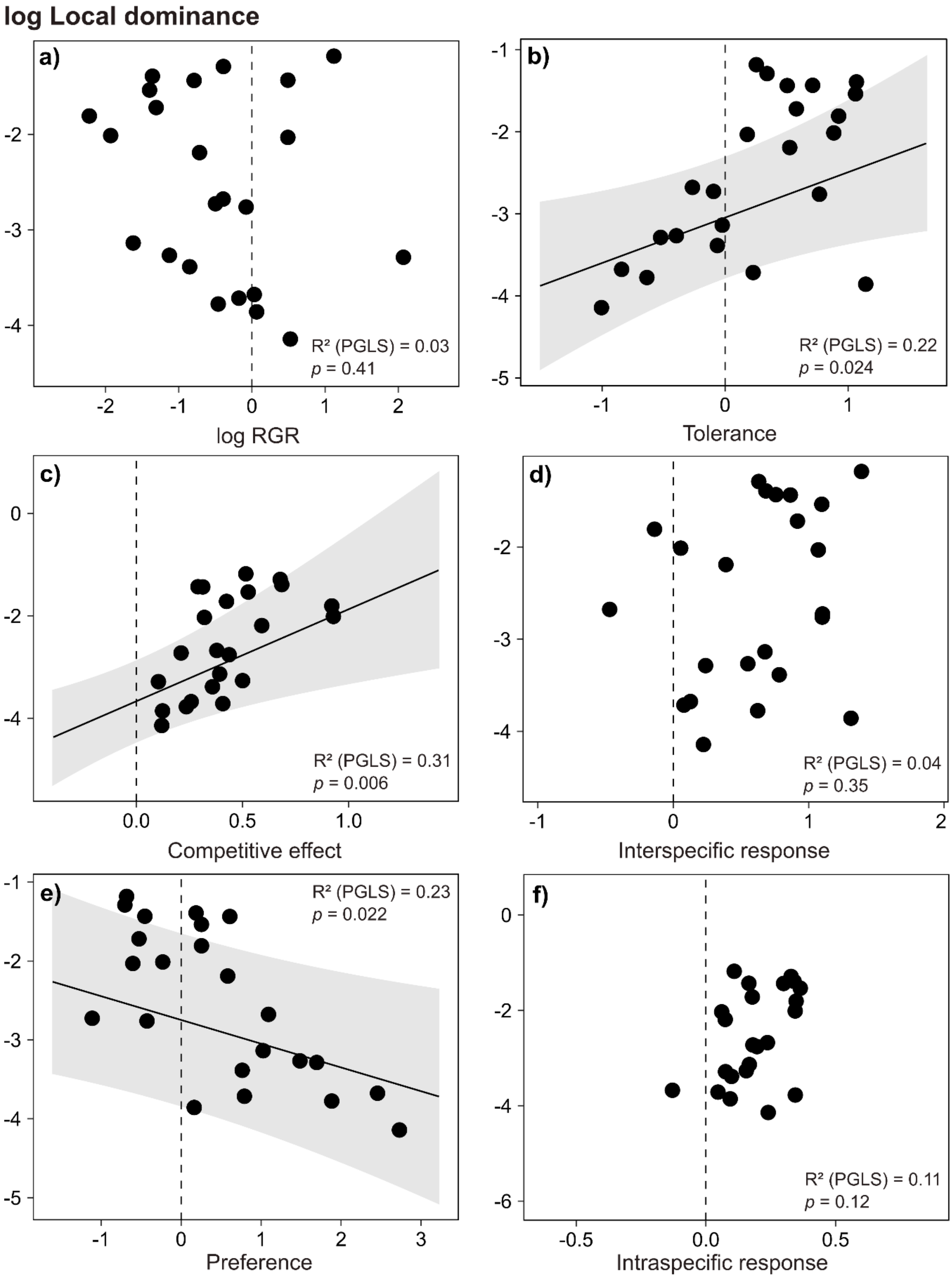

Fig. S12. Relationships between local dominance and metrics of abiotic tolerance and competitive ability in 23 wetland plant species. Lines are phylogenetic generalised least-squares (PGLS) fits with 95% confidence bands.

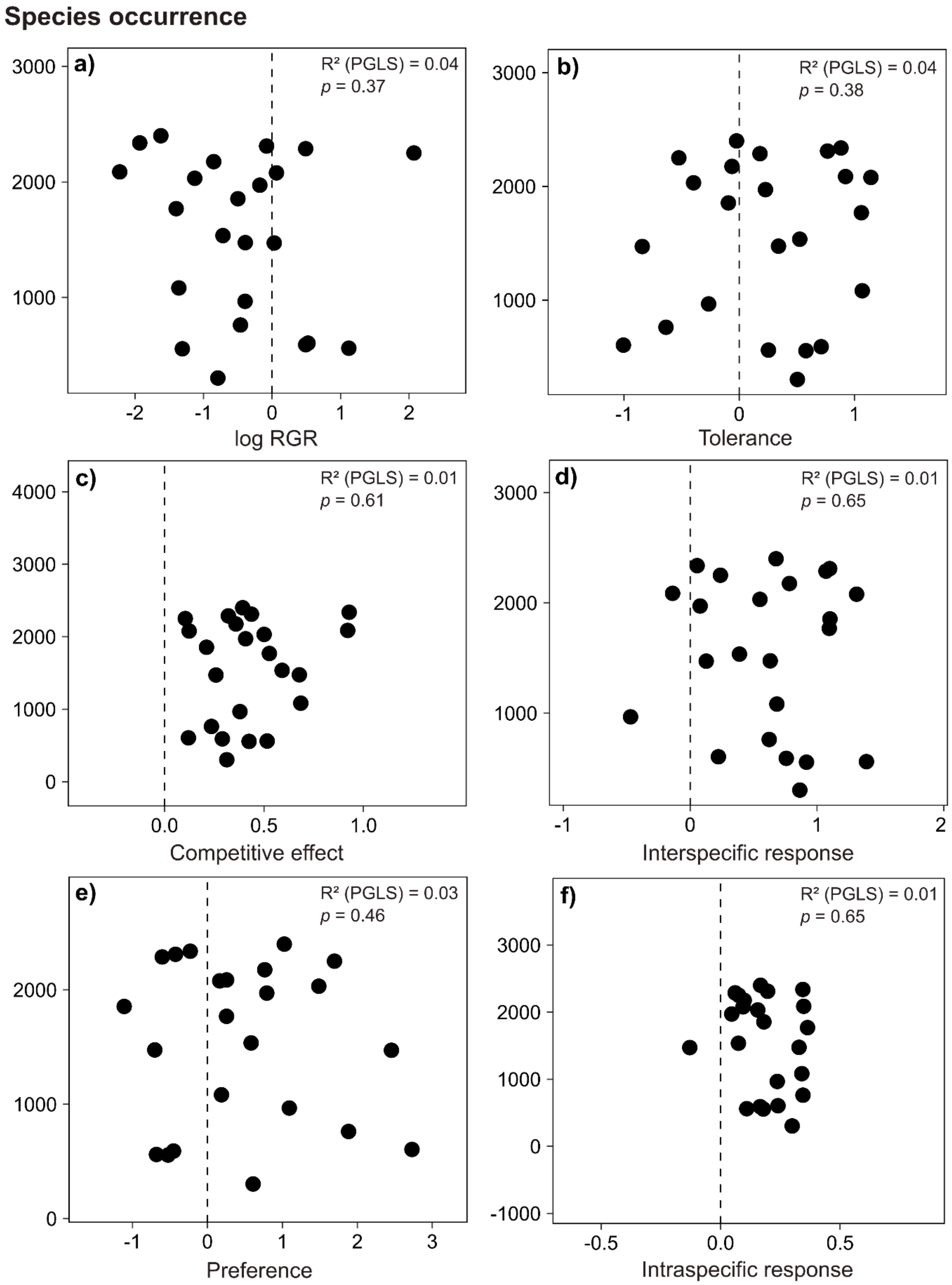

Fig. S13. Relationships between species occurrence and metrics of abiotic tolerance and competitive ability in 23 wetland plant species. The y-axis represents the total number of species occurrences across 2551 grid cells covering the territory of the Czech Republic.

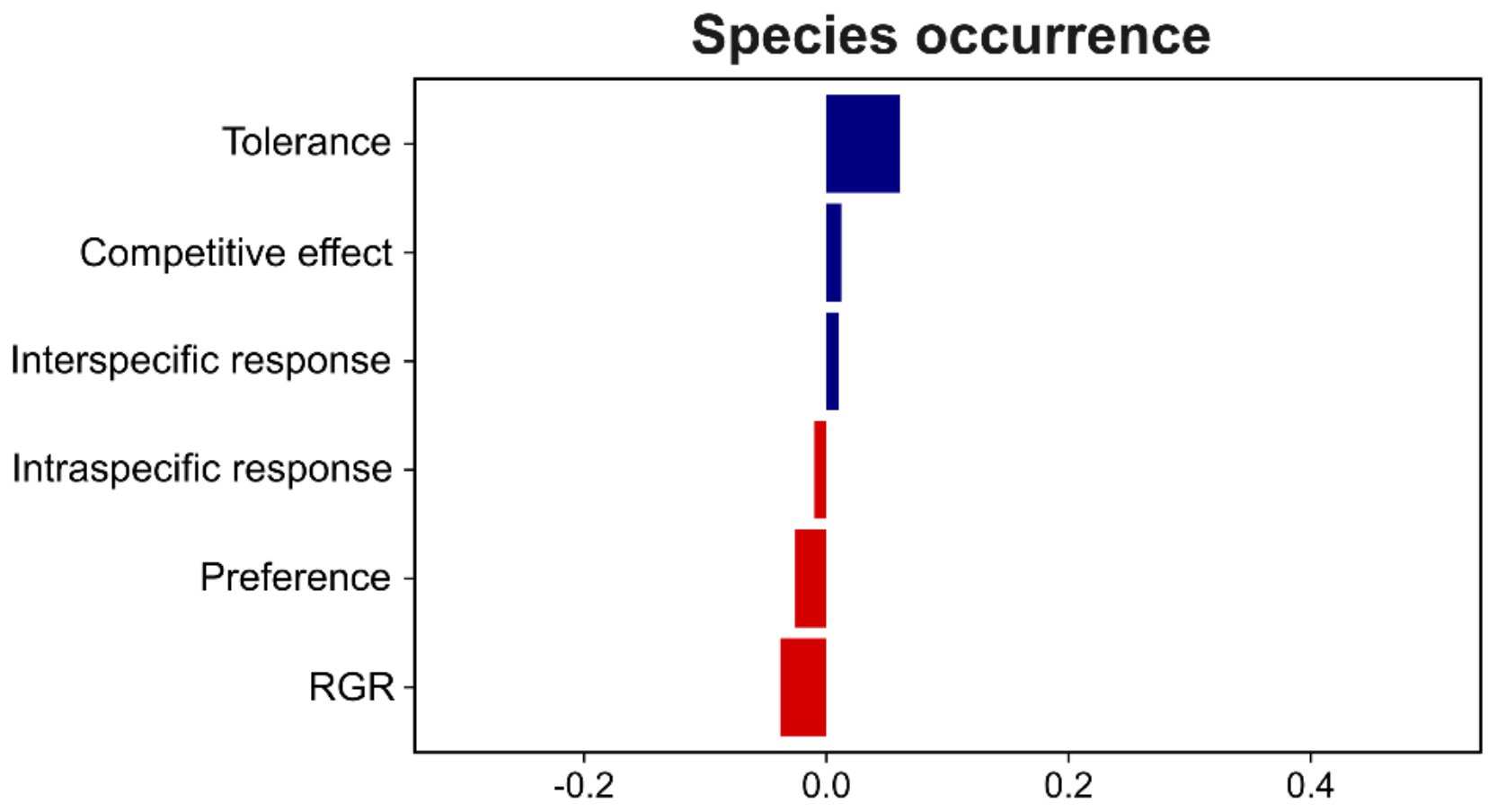

Fig. S14. Species occurrence across 23 wetland plant species. Relationships of occurrence (occupied / total grid quadrants in the Czech Republic CEBA distribution grid; Wild et al. 2019) with six predictors. Bars show the signed coefficient of determination (R^2) from single-predictor PGLS models, with the sign taken from the model slope: blue = positive association, red = negative association. All relationships are non-significant.

Table S1. Study species with families (Stevens 2001 onwards), growth forms, total number of individuals per treatment, and number and percentage of individuals replanted during the establishment phase (percentages based on the total number of planted and replanted individuals).

Species	Family	Growth form	Treatment				No. ind.	
			Drought	High-water	Competition	Total	Replanted	Repl. (%)
Carex acuta	Cyperaceae	Sedge-like	7	9	45	61	4	6
Carex acutiformis	Cyperaceae	Sedge-like	7	10	53	70	9	11
Carex elata	Cyperaceae	Sedge-like	5	11	52	68	5	7
Carex elongata	Cyperaceae	Sedge-like	5	4	28	37	39	51
Carex paniculata	Cyperaceae	Sedge-like	7	10	54	71	10	12
Carex pseudocyperus	Cyperaceae	Sedge-like	7	8	46	61	11	15
Carex remota	Cyperaceae	Sedge-like	6	9	28	43	23	35
Carex riparia	Cyperaceae	Sedge-like	2	3	10	15	1	6
Cirsium palustre	Asteraceae	Forb	6	6	21	33	40	55
Deschampsia cespitosa	Poaceae	Grass	6	9	58	73	4	5
Epilobium hirsutum	Onagraceae	Forb	6	10	49	65	24	27
Glyceria fluitans	Poaceae	Grass	7	9	48	64	9	12
Glyceria maxima	Poaceae	Grass	6	8	41	55	8	13
Iris pseudacorus	Iridaceae	Forb	6	8	52	66	11	14
Juncus effusus	Juncaceae	Sedge-like	6	8	29	43	11	20
Lysimachia vulgaris	Primulaceae	Forb	5	4	19	28	50	64
Lythrum salicaria	Lythraceae	Forb	6	8	43	57	36	39
Myosotis palustris agg.	Boraginaceae	Forb	6	8	43	57	38	40
Phalaris arundinacea	Poaceae	Grass	5	8	44	57	2	3
Poa trivialis	Poaceae	Grass	6	9	41	56	5	8
Rumex conglomeratus	Polygonaceae	Forb	6	9	50	65	1	2
Rumex sanguineus	Polygonaceae	Forb	6	8	43	57	6	10
Scirpus sylvaticus	Cyperaceae	Sedge-like	2	2	15	19	38	67

Table S2. Results of species-specific linear mixed models used to predict above-ground biomass from morphological traits. Because whole-plant biomass could not be measured non-destructively, above-ground biomass was estimated from traits known to correlate with biomass (number of ramets, number of branches, number of leaves, plant height, and leaf length; see Table SX). Intercept and slope coefficients are shown for each species; significance levels are indicated ($P < 0.05$, $P < 0.01$, $P < 0.001$). “EX” denotes excluded predictors, and “n.a.” predictors not measured for a given species. R^2m and R^2c represent marginal and conditional coefficients of determination, respectively (Nakagawa and Schielzeth 2013).

Species	Intercept	No. of ramets	No. of branches	No. of leaves	Plant height	Leaf length	Random effect	R^2_m	R^2_c
Carex acuta	0.24	0.045***	n.a.	n.a.	0.018**	0.014*	Included	0.51	0.73
Carex acutiformis	0.34	0.074***	n.a.	n.a.	EX	0.031***	Included	0.47	0.66
Carex elata	–0.66	0.067***	n.a.	n.a.	0.023**	0.025**	Included	0.59	0.78
Carex elongata	–1.88***	0.029***	n.a.	n.a.	0.102***	EX	Included	0.81	0.85
Carex paniculata	–1.93***	0.093***	n.a.	n.a.	0.017**	0.030***	Included	0.87	0.95
Carex pseudocyperus	0.19	0.085***	n.a.	n.a.	0.032***	EX	Included	0.72	0.86
Carex remota	–0.67**	0.017***	n.a.	n.a.	EX	0.049***	Included	0.74	0.97
Carex riparia	–2.67***	0.250***	n.a.	n.a.	EX	0.070***	Included	0.88	0.93
Cirsium palustre	–2.06***	n.a.	n.a.	0.095***	EX	0.102***	Included	0.77	0.93
Deschampsia cespitosa	0.64	0.011***	n.a.	n.a.	0.027**	EX	Included	0.60	0.71
Epilobium hirsutum	–0.83**	0.161***	EX	n.a.	0.025***	0.233***	Included	0.55	0.86
Glyceria fluitans	0.24	0.011***	n.a.	n.a.	0.020***	0.053***	Included	0.58	0.77
Glyceria maxima	–1.74**	0.058***	n.a.	n.a.	0.042***	0.021	Included	0.66	0.80
Species	Intercept	No. of ramets	No. of branches	No. of leaves	Plant height	Leaf length	Random effect	R^2_m	R^2_c
Iris pseudacorus	–1.50***	n.a.	n.a.	0.049***	EX	0.061***	Included	0.69	0.85

Juncus effusus	–1.24***	0.012***	n.a.	n.a.	EX	0.063***	Included	0.71	0.88
Lysimachia vulgaris	–4.47***	EX	EX	n.a.	EX	0.66***	Included	0.81	0.98
Lythrum salicaria	–0.93**	n.a.	0.073***	n.a.	0.047***	EX	Included	0.66	0.86
Myosotis palustris agg.	–1.11**	0.053***	n.a.	n.a.	0.090*	EX	Not included	0.84	0.84
Phalaris arundinacea	0.93**	0.023***	n.a.	n.a.	0.015***	EX	Included	0.55	0.66
Poa trivialis	0.05	0.012***	n.a.	n.a.	EX	0.044	Included	0.47	0.84
Rumex conglomeratus	–1.46***	n.a.	n.a.	0.036***	EX	0.110***	Included	0.68	0.83
Rumex sanguineus	–2.17***	n.a.	n.a.	0.040***	EX	0.140***	Included	0.77	0.85
Scirpus sylvaticus	–1.83*	0.037**	n.a.	n.a.	0.094***	EX	Included	0.77	1.00

Table S3. Functional traits measured in this study across three experimental treatments (drought, high-water, high-water competition). Values are species-level medians with 50% confidence intervals. The column *p* indicates the significance of treatment effects based on a linear mixed-effects model with species identity included as a random factor. When the assumption of residual normality was violated, variables were log-transformed prior to analysis.

Organ	Trait	Units	Drought	High-water	High-water competition	*p*
Leaf	Carbon isotope ratio ($\delta^{13}C$)	$\delta^{13}C$	−28.56 [−29.95, −27.71]	−28.01 [−29.10, −27.74]	−28.39 [−29.37, −28.00]	***
	Leaf carbon concentration (Leaf C)	mg g^{-1}	42.97 [41.77, 43.76]	42.59 [40.50, 43.24]	43.16 [41.86, 43.55]	ns
	Leaf dry matter content (LDMC)	%	258.0 [197.6, 302.0]	259.4 [183.2, 302.1]	268.8 [225.0., 291.0]	ns
	Leaf nitrogen concentration (Leaf N)	%	1.97 [1.72, 2.08]	1.67 [1.56, 1.90]	1.65 [1.56, 1.91]	***
	Leaf phosphorus concentration (Leaf P)	mg kg^{-1}	3200 [2653, 3587]	2423 [1803, 2968]	2542 [1868, 2848]	***
	Specific leaf area (SLA)	cm^2 g^{-1}	196.8 [161.8, 219.9]	179.1 [143.7, 216.7]	187.6 [160.5, 217.9]	ns
	Stomatal conductance	mmol m^{-2} s^{-1}	471 [344, 639]	429 [358, 489]	426 [350, 509]	***
	Stomatal fraction	%	6.8 [4.7, 10.5]	7.0 [5.3, 11.5]	7.2 [4.6, 10.4]	ns
Root	Root specific volume (RSV)	mL mg^{-1}	0.98 [0.91, 1.17]	1.19 [0.96, 1.31]	1.25 [1.04, 1.35]	***
	Maximum root length (MRL)	cm	51 [47, 57]	37 [23, 48]	38 [25, 48]	***
Whole plant	Plant height	cm	38.5 [19.3, 44.5]	36.8 [18.0, 46.0]	35.5 [17.5, 45.3]	***
	Relative growth rate (RGR)	g day^{-1}	0.65 [0.23, 1.22]	0.44 [0.27, 0.70]	0.63 [0.31, 1.15]	***
	Root mass fraction (RMF)	g g^{-1}	0.49 [0.34, 0.63]	0.42 [0.33, 0.55]	0.45 [0.34, 0.57]	***

Table S4. Comparison of phylogenetic generalized least squares (PGLS) models explaining species competitive effect, interspecific response, and intraspecific response. For each response variable, three candidate models were fitted using preference and tolerance as predictors: a model including preference only, a model including tolerance only, and a model including both predictors. Models are ranked by the corrected Akaike information criterion (AICc). The dashed line marks the boundary between models with substantial support (ΔAICc < 2) and lower-ranked models. Variance inflation factor was 2.0, indicating no severe multicollinearity among predictors. Estimated regression coefficients are provided for the intercept and for predictors included in a given model, whereas EX indicates that the predictor was excluded. Asterisks denote predictor significance within models (*, $P < 0.05$; **, $P < 0.01$).

Response variable	Rank	Intercept	Preference	Tolerance	AICc	ΔAICc
Competitive effect ~	1.	0.359	EX	0.193**	-9.392	0
	2.	0.345	0.015	0.203*	-6.827	2.565
	3.	0.426	-0.055	EX	-3.676	5.716
Interspecific response ~	1.	0.743	-0.262**	EX	28.489	0
	2.	0.775	-0.292*	-0.071	31.018	2.528
	3.	0.548	EX	0.273	33.955	5.466
Intraspecific response ~	1.	0.123	EX	0.103*	-32.310	0
	2.	0.084	0.039	0.135**	-31.753	0.557
	3.	0.150	-0.004	EX	-27.684	4.625

Table S5. Pairwise Pearson correlations (r) among functional traits. Strong correlations ($|r| \geq 0.6$) are shown in bold. Asterisks denote significance levels after Benjamini–Hochberg correction for multiple testing (*, $P < 0.05$; **, $P < 0.01$; ***, $P < 0.001$).

	Stomatal conductance	δ¹³C	Leaf P	RMF	Leaf N	Stomatal fraction	SLA	RSV	Leaf C	LDMC	Root length	Plant height
Stomatal conductance	–											
δ¹³C	**−0.66****	–										
Leaf P	0.10	−0.05	–									
RMF	0.34	−0.24	−0.29	–								
Leaf N	−0.09	0.14	0.22	0.41	–							
Stomatal fraction	−0.28	0.29	−0.20	0.03	0.31	–						
SLA	−0.32	−0.03	0.29	0.05	0.10	−0.29	–					
RSV	−0.04	0.39	0.05	−0.04	0.07	**0.65****	−0.47	–				
Leaf C	−0.26	0.35	−0.27	0.15	0.50	**0.57***	−0.32	0.40	–			
LDMC	−0.37	0.48	−0.12	−0.15	0.40	0.42	−0.20	0.25	**0.87*****	–		
MRL	0.19	0.17	0.00	0.14	0.27	0.38	−0.42	**0.62***	**0.62***	**0.57***	–	
Plant height	0.07	0.03	−0.15	0.20	0.18	0.31	−0.24	0.35	**0.71****	**0.70****	**0.78*****	–

Table S6. Pairwise phylogenetically corrected Pearson correlations (r) among functional traits. Strong correlations ($|r| \geq 0.6$) are shown in bold. Asterisks denote significance levels after Benjamini–Hochberg correction for multiple testing (*, $P < 0.05$; **, $P < 0.01$; ***, $P < 0.001$).

	Stomatal conductance	δ¹³C	Leaf P	RMF	Leaf N	Stomatal fraction	SLA	RSV	Leaf C	LDMC	Root length	Plant height
Stomatal conductance	–											
δ¹³C	**−0.72****	–										
Leaf P	0.49	−0.36	–									
RMF	0.52	**−0.67****	−0.02	–								
Leaf N	0.45	**−0.58***	0.51	0.49	–							
Stomatal fraction	0.32	−0.51	−0.11	**0.68****	0.21	–						
SLA	−0.43	0.15	0.25	−0.14	0.13	−0.25	–					
RSV	0.26	−0.19	0.23	0.17	0.04	0.28	−0.29	–				
Leaf C	0.30	−0.05	−0.17	0.29	0.22	0.28	−0.47	−0.18	–			
LDMC	0.12	0.07	−0.22	0.14	0.28	0.18	−0.42	−0.19	**0.75****	–		
MRL	**0.73****	**−0.56***	0.15	**0.63***	0.33	**0.60***	−0.46	0.49	0.35	0.34	–	
Plant height	0.38	−0.37	−0.16	**0.67****	0.16	**0.56***	−0.42	0.23	0.49	0.52	**0.77****	–

Table S7. All-subsets comparison of phylogenetic generalized least squares (PGLS) models explaining species niche breadth (θ). Candidate models were constructed from all possible combinations of six predictor variables: tolerance, competitive effect, interspecific response, preference, intraspecific response, and RGR, yielding 64 candidate models in total. Models are ranked by the corrected Akaike information criterion (AICc), and the table presents the 15 highest-ranked models. Each row represents one candidate model. Estimated regression coefficients are provided for predictors included in the model, whereas EX indicates that the predictor was excluded. The dashed line marks the boundary between models with substantial support (ΔAICc < 2) and lower-ranked models. Asterisks denote predictor significance within models (**, $P < 0.01$; ***, $P < 0.001$). Variance inflation factors for the global model ranged from 1.46 to 3.09, indicating no severe multicollinearity among predictors.

Rank	Intercept	Comp. eff.	Inter. resp.	Intra. resp.	Prefer.	RGR	Toler.	AICc	ΔAICc
1.	5.39	1.62***	EX	EX	EX	EX	EX	21.076	0
2.	5.21	1.82***	0.234	EX	EX	EX	EX	22.122	1.046
3.	5.35	1.77***	EX	EX	EX	0.056	EX	23.356	2.280
4.	5.21	1.69***	EX	EX	0.125	EX	EX	23.371	2.295
5.	5.34	1.74***	EX	EX	EX	EX	-0.092	23.429	2.353
6.	5.39	1.63***	EX	-0.007	EX	EX	EX	23.739	2.663
7.	4.96	2.02***	0.326	EX	0.131	EX	EX	24.046	2.970
8.	5.10	2.03***	0.273	EX	EX	EX	-0.153	24.240	3.164
9.	5.20	1.88***	0.216	EX	EX	0.031	EX	24.949	3.873
10.	5.21	1.81***	0.235	0.036	EX	EX	EX	25.078	4.002
11.	5.12	2.00***	EX	EX	0.136	0.097	EX	25.326	4.250
12.	5.31	1.87**	EX	EX	EX	0.051	-0.084	26.069	4.993
13.	5.21	1.76***	EX	EX	0.098	EX	-0.077	26.149	5.073
14.	5.22	1.74***	EX	-0.189	0.124	EX	EX	26.248	5.172
15.	5.34	1.75**	EX	0.150	EX	0.062	EX	26.271	5.195

Table S8. All-subsets comparison of phylogenetic generalized least squares (PGLS) models explaining species log local dominance. Candidate models were constructed from all possible combinations of six predictor variables: tolerance, competitive effect, interspecific response, preference, intraspecific response, and RGR, yielding 64 candidate models in total. Models are ranked by the corrected Akaike information criterion (AICc), and the table presents the 15 highest-ranked models. Each row represents one candidate model. Estimated regression coefficients are provided for predictors included in the model, whereas EX indicates that the predictor was excluded. The dashed line marks the boundary between models with substantial support (ΔAICc < 2) and lower-ranked models. Asterisks denote predictor significance within models (^{m}, $P < 0.1$; *, $P < 0.05$; **, $P < 0.01$; ***, $P < 0.001$). Variance inflation factors for the global model ranged from 1.46 to 3.09, indicating no severe multicollinearity among predictors.

Rank	Intercept	Comp. eff.	Inter. resp.	Intra. resp.	Prefer.	RGR	Toler.	AICc	ΔAICc
1.	-3.61	1.70*	EX	EX	EX	0.317*	0.443^{m}	41.063	0
2.	-3.51	1.99**	EX	EX	EX	0.201	EX	41.579	0.516
3.	-3.31	1.53*	EX	EX	EX	EX	EX	41.710	0.647
4.	-3.81	2.31**	EX	EX	0.125	0.262*	EX	42.010	0.947
5.	-4.10	2.58***	0.348	EX	EX	0.218^{m}	EX	42.271	1.2087
6.	-3.87	1.86**	EX	1.85	EX	0.355*	EX	42.305	1.242
7.	-3.69	1.44*	EX	1.45	0.131	0.387**	0.386	42.488	1.425
8.	-4.16	2.14**	0.340	1.84	EX	0.304*	EX	42.592	1.529
9.	-4.04	2.18**	0.445^{m}	EX	EX	EX	EX	42.861	1.798
10.	-3.45	1.60*	EX	EX	EX	0.263*	0.331	43.024	1.961
11.	-3.82	1.99*	0.199	EX	0.136*	0.276*	0.346	43.530	2.468
12.	-2.81	EX	-0.306	2.91	EX	0.310*	EX	43.650	2.587
13.	-3.09	EX	EX	2.44	0.098*	0.320*	0.339	43.701	2.639
14.	-3.48	1.19^{m}	EX	1.81	0.124^{m}	0.334*	0.233	43.814	2.751
15.	-3.25	EX	EX	2.10	EX	0.389**	0.581*	44.068	3.005

Supporting References

Chytrý, M. (Ed.). (2011). *Vegetation of the Czech Republic 3: Aquatic and wetland vegetation*. Academia.

Chytrý, M. & Rafajová, M. (2003). Czech National Phytosociological Database: basic statistics of the available vegetation-plot data. *Preslia* 75: 1–15.

Farquhar, G. D., Ehleringer, J. R. & Hubick, K. T. (1989). Carbon isotope discrimination and photosynthesis. *Annual Review of Plant Physiology and Plant Molecular Biology* 40: 503–537.

Fridley, J. D., Vandermast, D. B., Kuppinger, D. M., Manthey, M., & Peet, R. K. (2007). Co-occurrence based assessment of habitat generalists and specialists: A new approach for the measurement of niche width. *Journal of Ecology* 95: 707–722.

Gilbert, M. E., & Medina, V. (2016). Drought adaptation mechanisms should guide experimental design. *Trends in Plant Science* 21: 639–647.

Hulík, J., & Douda, J. (2017). Germination strategies of two dominant *Carex* species in a swamp alder forest: implications for restoration. *Biologia* 72: 370–377.

McCune B. & Mefford M. J. (1999) PC-ORD: multivariate analysis of ecological data 4.17. User's guide. – MjM Software, Gleneden Beach, OR, US.

McDowell, N., Pockman, W. T., Allen, C. D., Breshears, D. D., Cobb, N., Kolb, T., ... & Yepez, E. A. (2008). Mechanisms of plant survival and mortality during drought: why do some plants survive while others succumb to drought? *New Phytologist* 178: 719–739.

Nakagawa, S., & Schielzeth, H. (2013). A general and simple method for obtaining R^2 from generalized linear mixed-effects models. *Methods in Ecology and Evolution* 4: 133–142.

Stevens, P. F. (2001 onwards). Angiosperm Phylogeny Website. Version 14, July 2017 [and more or less continuously updated since].

Wild, J., Kaplan, Z., Danihelka, J., Petřík, P., Chytrý, M., Novotný, P., ... & Zouhar, V. (2019). Plant distribution data for the Czech Republic integrated in the Pladias database. *Preslia* 91: 1–24.

Zelený, D., & Chytrý, M. (2019). Ecological specialization indices for species of the Czech flora. *Preslia* 91: 93–116.
